# Position Statement of the Brazilian Society of Nephrology on incremental dialysis

**DOI:** 10.1590/2175-8239-JBN-2025-0374en

**Published:** 2026-06-26

**Authors:** Fernanda Salomão Gorayeb-Polacchini, Thyago Proença de Moraes, Rosilene Motta Elias, Allison Andrade, Dirceu Reis da Silva, Felipe Costa Neves, Fernando das Mercês de Lucas, Isadora Cartaxo de Sousa Calvo, Juliana El Ghoz Leme, Maria Gabriela Guimarães, Pasqual Barretti, Patrícia Ferreira Abreu, Paulo Henrique Santos Fraxino, Ramon Lima, Stenio Barbosa de Freitas, Viviane Calice-Silva, José Andrade Moura-Neto

**Affiliations:** 1 Sociedade Brasileira de Nefrologia, São Paulo, SP, Brazil.; 2 Hospital de Base, FUNFARME, São José do Rio Preto, SP, Brazil.; 3 Pontifícia Universidade Católica do Paraná, Curitiba, PR, Brazil.; 4 Nefroclínicas Curitiba, PR, Brazil.; 5 Universidade de São Paulo, São Paulo, SP, Brazil.; 6 Universidade Nove de Julho, São Paulo, SP, Brazil.; 7 Hospital de Clínicas de Porto Alegre, Porto Alegre, RS, Brazil.; 8 Instituto de Doenças Renais, Porto Alegre, RS, Brazil.; 9 UNISINOS, São Leopoldo, RS, Brazil.; 10 Escola Bahiana de Medicina e Saúde Pública, Salvador, BA, Brazil.; 11 Hospital Ana Nery, Departamento de Nefrologia, Salvador, BA, Brazil.; 12 Universidade Federal da Bahia, Programa de Pós-Graduação em Ciências da Saúde, Salvador, BA, Brazil.; 13 International Renal Research Institute of Vicenza, Vicenza, Italia.; 14 Universidade Estadual Paulista, Faculdade de Medicina de Botucatu, Botucatu, São Paulo, Brazil.; 15 Universidade Federal de São Paulo, São Paulo, SP, Brazil.; 16 Hospital São João de Deus, Divinópolis, MG, Brazil.; 17 Fundação Pró-rim, Joinville, SC, Brazil.; 18 Universidade da Região de Joinville, Joinville, SC, Brazil.

**Keywords:** Renal Dialysis, Hemodialysis, Peritoneal Dialysis, Chronic Kidney Failure, Renal Replacement Therapy, Quality of Life, Incremental Dialysis, Consensus

## Abstract

Incremental dialysis, applicable to both hemodialysis (HD) and peritoneal dialysis (PD), is an individualized approach. It consists of offering a dialysis dose adjusted to the patient’s residual renal function (RRF) in order to achieve the same clinical outcomes observed with full doses, while improving quality of life and reducing exposure to the risks associated with the dialysis procedure. This Position Statement of the Brazilian Society of Nephrology (SBN) aims to present recommendations on eligibility criteria, prescription, monitoring, and safe implementation, as well as to report the clinical results already described with this approach. Eligibility includes a residual diuresis ≥ 500 mL/24h in HD or ≥ 100 mL/24h in PD and/or a urea clearance (Kru) ≥ 2.0 mL/min/1.73 m^2^, as well as clinical stability and adequate volume and metabolic control. Monitoring with regular reassessment of RRF is recommended. Indicators for dialysis dose intensification include hypervolemia, uremic symptoms, worsening of nutritional status, hyperkalemia, hyperphosphatemia, refractory metabolic acidosis, and laboratory findings of subdialysis. The implementation of incremental dialysis requires well-defined institutional protocols, systematic education of patients and families, a properly trained multidisciplinary team, and a shared decision-making process. Scientific evidence suggests that incremental dialysis is safe and effective, attenuating the loss of RRF, and can reduce hospitalizations, while maintaining or improving quality of life, without increasing mortality. Additionally, it may contribute to cost reduction and greater sustainability of the healthcare system, and should be considered an integral part of the contemporary therapeutic armamentarium.

## Introduction

The prescription of dialysis based on urea kinetics (Kt/V) was established in the 1980s and has been consolidated as a standard in patients with chronic kidney disease on dialysis (CKD-5D)^1,2^. Traditionally, this model defines a uniform prescription, centered on the frequency and duration of sessions, without considering individual variations in residual renal function (RRF).

In 1985, Bonomini and colleagues introduced the concept of incremental dialysis, proposing to adjust the intensity of treatment according to RRF^3^. This approach recognizes that, at the beginning of dialysis therapy, between 30% and 70% of patients maintain significant RRF, whose contribution to overall clearance is often underestimated^4,5^. RRF is defined by the presence of a 24-hour diuresis ≥ 100 mL or a urea clearance (Kru) ≥ 2.0 mL/min/1.73 m^2 5,6^. Its maintenance is clinically relevant, since it contributes to the removal of higher-molecular-weight uremic toxins and to the maintenance of fluid, electrolyte, and metabolic homeostasis^6,7^.

Conventional hemodialysis (HD) is defined as three to four sessions per week, lasting three to five hours each. In practice, the three-times-weekly regimen, lasting four hours per session, prevails—a regimen that has been historically established and has demonstrated efficacy in improving survival and providing clinical and metabolic stability. However, initiating full-dose dialysis treatment in patients with preserved RRF may represent unnecessary exposure to the risks inherent to the therapy and its complications, in addition to potentially accelerating the loss of RRF. Kidney Disease Outcomes Quality Initiative (KDOQI) guidelines recommend that HD patients with Kru < 2.0 mL/min/1.73 m^2^ receive a minimum of three sessions per week, with at least three hours per session^5^. Although it aims to ensure minimum dialysis adequacy, this recommendation does not necessarily reflect the individual needs of patients with preserved RRF.

Currently, dialysis adequacy should not be defined solely by Kt/V, but should also incorporate complementary indicators, such as RRF, clinical and laboratory conditions, quality of life, and patient preferences. The incremental model integrates residual renal clearance with dialysis to compose the total clearance, targeting a weekly Kt/V *(*Kt/V standard-stdKt/V*)* compatible with clinical and metabolic adequacy. Thus, incremental dialysis replaces the uniform prescription paradigm with an individualized, dynamic, and patient-centered approach, with a gradual transition to the conventional regimen as RRF declines^5,8,9^.

In practice, many patients start dialysis in an emergency context, often with uremia, fluid and electrolyte disorders, volume overload, anemia, and alterations in mineral and bone metabolism. In these cases, it is appropriate to start with a full dose of dialysis, aiming at clinical stabilization and correction of metabolic alterations. After stabilization of the condition and adequate evaluation of RRF, a progressive reduction in the intensity of treatment can be considered, provided that strict safety and monitoring criteria are maintained. It is essential to make this dynamic approach explicit to avoid the misinterpretation that incremental dialysis should be adopted immediately and universally, reinforcing its individualized nature and dependence on the clinical context.

In PD, the concept of the incremental approach is more consolidated, with its conceptualization and recommendation dating back to 1997^10^. Since 2006, the International Society for Peritoneal Dialysis (ISPD) has recommended initiating therapy with partial PD regimens, gradually increasing the number of exchanges as RRF declines^11^. Evidence indicates that the incremental approach to PD has mortality similar to that of conventional regimens, with greater preservation of RRF, better quality of life, reduced exposure to bioincompatible solutions, lower risk of peritonitis, and reduced resource utilization and treatment-related costs^12,13^.

In HD, the incremental approach has received increasing attention in recent years. Studies show that the strategy is safe and effective, being associated with better preservation of RRF, lower levels of inflammation, and reduced hospitalizations and healthcare costs^14,15,16,17,18,19,20^, with mortality similar to that observed in conventional HD, provided that eligibility and monitoring criteria are established^17,20,21,22^.

The clinical relevance of RRF preservation is widely documented. Evidence suggests that starting dialysis with conventional or intensive regimens may accelerate the loss of RRF, possibly due to recurrent episodes of intradialytic hypotension, hemodynamic fluctuations, and inflammatory activation^23,24^. Patients with preserved RRF have a lower cardiovascular risk, better nutritional status, improved control of anemia and volume overload, and better quality of life^24,25,26^. Thus, regardless of the dialysis modality, strategies should be adopted to preserve RRF, including adequate blood pressure and glycemic control, as well as avoidance of nephrotoxic agents.

In Brazil, the 2024 Dialysis Census of the Brazilian Society of Nephrology (SBN) reports well over 170 thousand individuals on dialysis therapy; however, despite the substantial number, the proportion of patients under an incremental regimen remains unknown^27^. Given the magnitude of the population on dialysis, the clinical heterogeneity of patients, and the need to optimize resources in the Unified Health System (SUS), an official position statement of the SBN on incremental dialysis is essential. This position seeks to establish eligibility criteria, monitoring parameters, and practical guidance for the safe implementation of this strategy, promoting advances in both the personalization of care and the sustainability of the health system.

## Methods

This document constitutes an official position statement of the SBN, prepared by a technical group composed of members of the Board of Directors, the Department of Dialysis, the Peritoneal Dialysis Committee, and the Patient Committee. The elaboration process included a narrative review and critical synthesis of the scientific literature.

The drafting of the recommendations was based on clinical consensus, and the final version of the document was reviewed and approved by all authors. It is, therefore, an institutional position statement of an educational nature, non-normative, whose objective is to guide clinical practice and promote standardization of practices related to incremental dialysis in the Brazilian context.

## Discussion

Incremental dialysis represents an individualized strategy for dialysis prescription, based on the use of the patient’s RRF. Unlike conventional regimens, the incremental approach proposes progressive and personalized adjustments that allow the achievement of clinical and metabolic outcomes comparable to those obtained with full-dose regimens, but with less exposure to dialysis^28^.

### Indications for Incremental Dialysis

The application of incremental dialysis requires well-defined clinical and laboratory criteria, as well as strict monitoring of safety parameters to reduce the risk of adverse events^11,29^. Key criteria include:

- **Residual renal function**: for PD patients, it is practically defined as a urine volume greater than 100 mL/24h^11,29^. In the case of HD, an incremental prescription is recommended when there is at least 500 mL of urine output in 24 hours^11,29,30^.

When 24-hour urine collection is not possible, RRF can be assessed by different measurement methods. The first is **home urine output recording**, in which the patient measures and records the urine volume produced in 24 hours using a graduated container and following the standardized guidance from the healthcare team. This method, although simple, provides a practical and relatively reliable estimate of RRF.

An alternative is the **serum measurement of beta-2 microglobulin**. Persistently elevated values (above 30 mg/L)—provided there are no infections, acute inflammatory processes, or conditions that may elevate this marker regardless of renal function—indicate a significant decline in RRF. In such cases, the incremental strategy is no longer appropriate, and adjustment to a conventional dialysis regimen should be considered^4^.

- **Symptoms**: absence of signs and/or symptoms associated with uremia.
- **Adequate control of volume status**: the patient should maintain fluid stability, with no recent episodes of hypervolemia or uncontrolled blood pressure attributed to the dialysis prescription. In HD, interdialytic weight gain should not exceed 5% of the estimated dry body weight between sessions^31^.
- **Treatment adherence and monitoring capacity**: motivated patients, with family or social support, and able to understand and follow the adapted dialysis schedule, as well as dietary and medication guidelines. It is essential that, from the outset, they are informed that this is a transitory strategy, subject to monthly adjustments according to clinical evaluation.
- **Clinical stability and absence of recent hospitalizations**: the patient must be clinically stable, with no recent hospitalizations due to volume decompensation, uremic symptoms, severe infection, or metabolic complications; contrarily, occasional hospitalizations not associated with the need for immediate adjustment of the dialysis dose do not contraindicate the incremental strategy. The frequency and causes for hospitalizations should be considered important parameters in the periodic evaluation of eligibility and safety for maintenance of the incremental regimen.
- **Anemia control**: the patient must have stable hemoglobin levels, without signs of severity or refractoriness, and with adequate response to treatment (erythropoiesis-stimulating agents, iron supplementation, and correction of deficiencies). The persistence of moderate to severe anemia, or the absence of response despite optimized management, should lead to reassessment of the prescription and eligibility for incremental dialysis, as well as investigation of secondary causes^11,29^.
- **Good nutritional status**: the patient must have an adequate nutritional status, with no evidence of hypercatabolism or significant protein-energy loss. There should be stability of nutritional laboratory parameters, as well as food intake compatible with dietary recommendations. The presence of malnutrition, inflammation, or marked catabolism should prompt specific nutritional intervention and reassessment of eligibility for incremental dialysis^11,29^.
- **Adequate potassium control**: the patient should have serum potassium levels within the normal range, with no recent clinically significant episodes of hyperkalemia. A serum potassium level ≤ 5.0 mEq/L is considered a safe parameter for maintaining the incremental strategy. Transient changes may occur but require assessment of dietary adherence, review of the dialysis prescription, and adjustment of medications that interfere with potassium homeostasis. In cases of recurrent or refractory hyperkalemia, intensification of the dialysis regimen should be considered. In the absence of other symptoms associated with uremia, pharmacological strategies for potassium control may be employed in conjunction with the incremental strategy^11,29^.
- **Adequate control of mineral and bone metabolism (CKD-MBD)**: the patient should have serum levels of parathyroid hormone (PTH), calcium, and phosphorus within the recommended ranges^11,29^.

### Monitoring of Dialysis Adequacy and Therapy Adjustments

Monitoring in incremental dialysis is continuous and requires periodic evaluation of the patient. This monitoring should be based on clinical and laboratory parameters. The frequency of assessment for each parameter is described in Chart 1 ^11,32,33,34^.

**Chart 1:** Proposal for minimal patient monitoring on incremental dialysis

Clinical-laboratory evaluation	Frequency of follow-up		
	Weekly	Monthly	Quarterly
Pre-dialysis blood pressure and intradialytic hypotension (hemodialysis patients)	X		
Clinical assessment of nutritional status		X	
Frailty and Karnofsky performance status			X
Quality of life and life expectancy			X
Psychological evaluation		X	
Residual urine output		X	
Residual renal function/urea clearance			X
Potassium and bicarbonate control		X	
Calcium and phosphorus control		X	
Parathyroid hormone and iron profile control			X
Anemia control		X	

Source: Adapted from Tangvoraphonkchai and Davenport, 2019.

- **Routine laboratory tests**: urea, creatinine, potassium, venous blood gas, calcium, phosphorus, PTH, hemoglobin, iron stores, and serum albumin.
  - In PD, total weekly Kt/V ≥ 1.7 (peritoneal + renal) should be measured; however, it is not indicated in isolation to define the dialysis dose. It is essential that the patient maintains well-being, clinical and laboratory control, adequate nutrition, and absence of volume overload.
  - For HD patients, RRF can be incorporated into the Kt/V calculation, reaching a weekly Kt/V ≥ 2.1, although this approach is not widely applied in clinical practice^2^. In addition, given the difficulty of reliably collecting 24-hour urine, especially in the elderly, serum beta-2 microglobulin measurement is suggested as an alternative for RRF assessment^14^.
- **Clinical evaluation**: presence of uremic symptoms, interdialytic weight gain (IDWG), frequency of hospitalizations, quality of life, and assessment of nutritional status.
- **Residual diuresis**: the simplest method to monitor residual diuresis is to ask the patient to measure 24-hour urine output once a month at home.

Incremental dialysis has the potential to delay the loss of RRF by reducing glucose exposure in PD and hemodynamic fluctuations in HD. However, RRF remains vulnerable to multiple acute and chronic factors, such as nephrotoxic drug use, contrast exposure, infections, and progression of the underlying disease.

The prescription of incremental dialysis should be dynamic and periodically reassessed, preferably during routine monthly consultations or earlier, when necessary. Monitoring of incremental dialysis should be documented in the medical record, including explicit justifications for maintaining the regimen when any parameter falls outside the established eligibility criteria, indicating possible adjustment and transition to conventional therapy.

Adjuvant measures to incremental dialysis may include, when appropriate, optimization of diuretic therapy (preferably loop diuretics), oral sodium bicarbonate, and the use of phosphorus and potassium binders, in addition to a diet with restricted potassium, phosphorus, and fluid intake, along with adequate protein consumption^4^. The most commonly used loop diuretic, furosemide, can be administered at doses of up to 240 mg/day. Although higher doses have been reported, their use should be judicious and accompanied by adequate monitoring due to the increased risk of ototoxicity^35,36,37,38^.

### Difference Between Incremental and Decremental Dialysis

**Figure 1:**
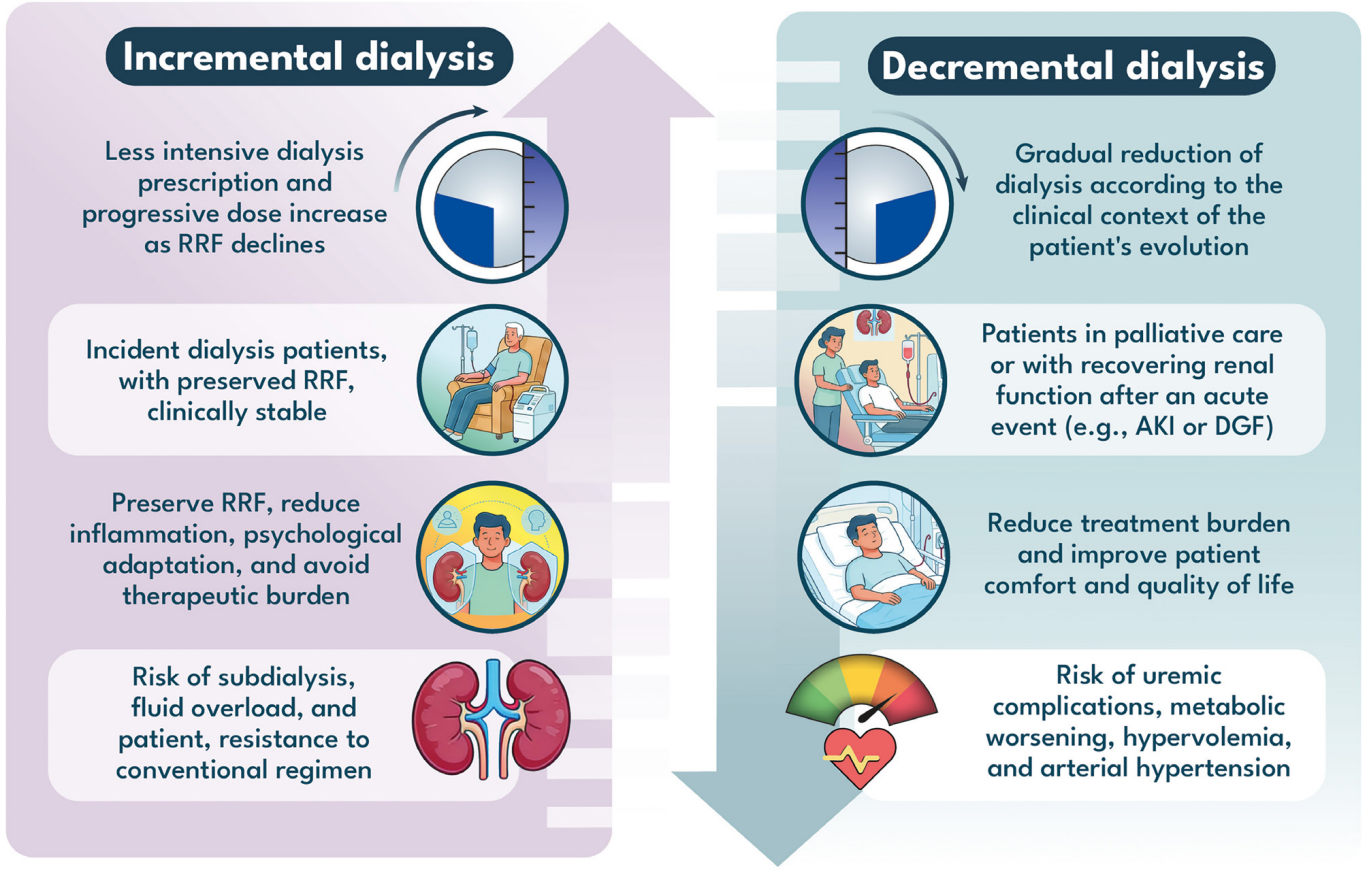
Comparison – incremental dialysis and decremental dialysis^8,9,21,31,39^.

Abbreviations: RRF, Residual Renal Function; AKI, Acute Kidney Injury; DGF, Delayed Graft Function.

While incremental dialysis is based on progressively increasing the intensity of dialysis as RRF is lost, decremental dialysis consists of the planned reduction of the dialysis dose in situations of partial recovery of renal function after acute kidney injury (AKI), improvement of delayed graft function (DGF), as well as in the context of palliative care. In these cases, the focus is on aligning treatment with the goals of comfort and quality of life^21^. Both models—incremental and decremental—are based on the same principle: to personalize therapy and adjust its intensity to the patient’s changing needs. The comparative analysis of these modalities is described in Figure 1. In this document, we will address incremental dialysis exclusively.

### Criteria for Transition to Conventional Dialysis Modality

Transition to conventional dialysis should occur when it is not possible to maintain clinical and laboratory parameters within the recommended targets, even after progressive dose adjustments in the incremental regimen. The indications are summarized in Chart 2.

**Chart 2:** Indications for transition to conventional dialysis

**Clinical:**
Signs/symptoms of hypervolemia and/or weight gain between sessions > 5% of dry body weight (in the case of hemodialysis).
Symptoms attributed to uremia.
Worsening of nutritional status.
Anuria.
Frequent hospitalization or hospitalization related to hypervolemia or uremia.
**Persistent laboratory changes for more than three months:**
Anemia refractory to the use of erythropoietin and/or iron supplementation and attributed to uremia.
Hyperphosphatemia and/or hyperkalemia refractory to dietary and pharmacological management.
Refractory metabolic acidosis.
Persistent serum beta-2 microglobulin (if available) > 30 mg/L in the absence of infection/inflammation.

With the decline of RRF, it is necessary to gradually increase the intensity of therapy until a full-dose regimen is reached:

- PD: Continuous Ambulatory Peritoneal Dialysis (CAPD), with four exchanges totaling 8 L/day, or Continuous Cycling Peritoneal Dialysis (CCPD) performed daily (i.e., Automated Peritoneal Dialysis – APD – overnight, added to a daytime manual exchange, totaling 24 hours of dialysis every day of the week);
- HD: three sessions per week, each lasting four hours.

During the transition from incremental to conventional dialysis, it is recommended to gradually adjust the duration and frequency of sessions in order to minimize symptoms and discomfort. Shorter HD sessions (two to three hours) may be adopted temporarily as the frequency is increased, with further adjustments based on the clinical response.

**Figure 2:**
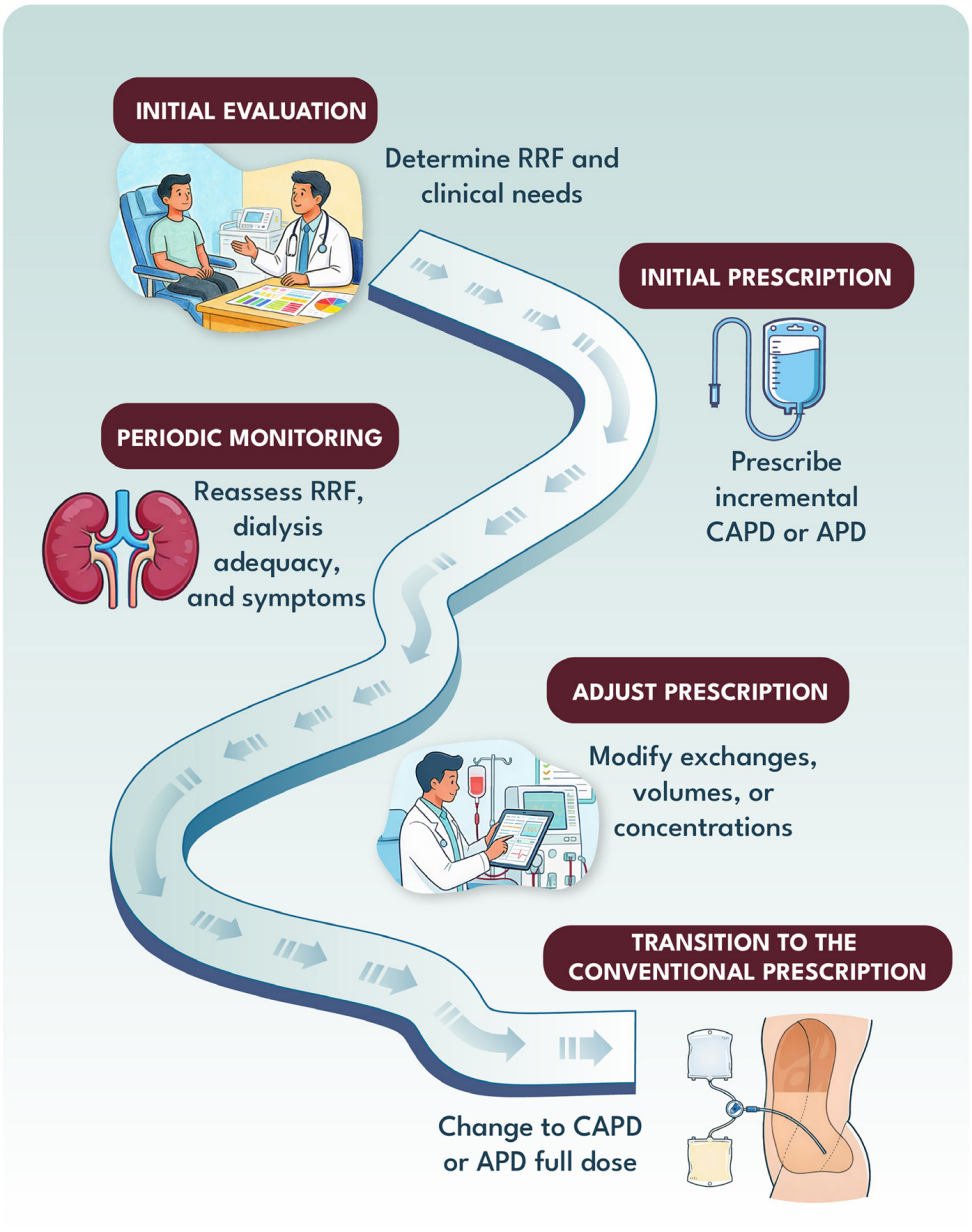
Schematic model of the patient’s trajectory on incremental peritoneal dialysis.

Abbreviations: RRF, Residual Renal Function; CAPD, Continuous Ambulatory Peritoneal Dialysis; APD, Automated Peritoneal Dialysis.

**Figure 3:**
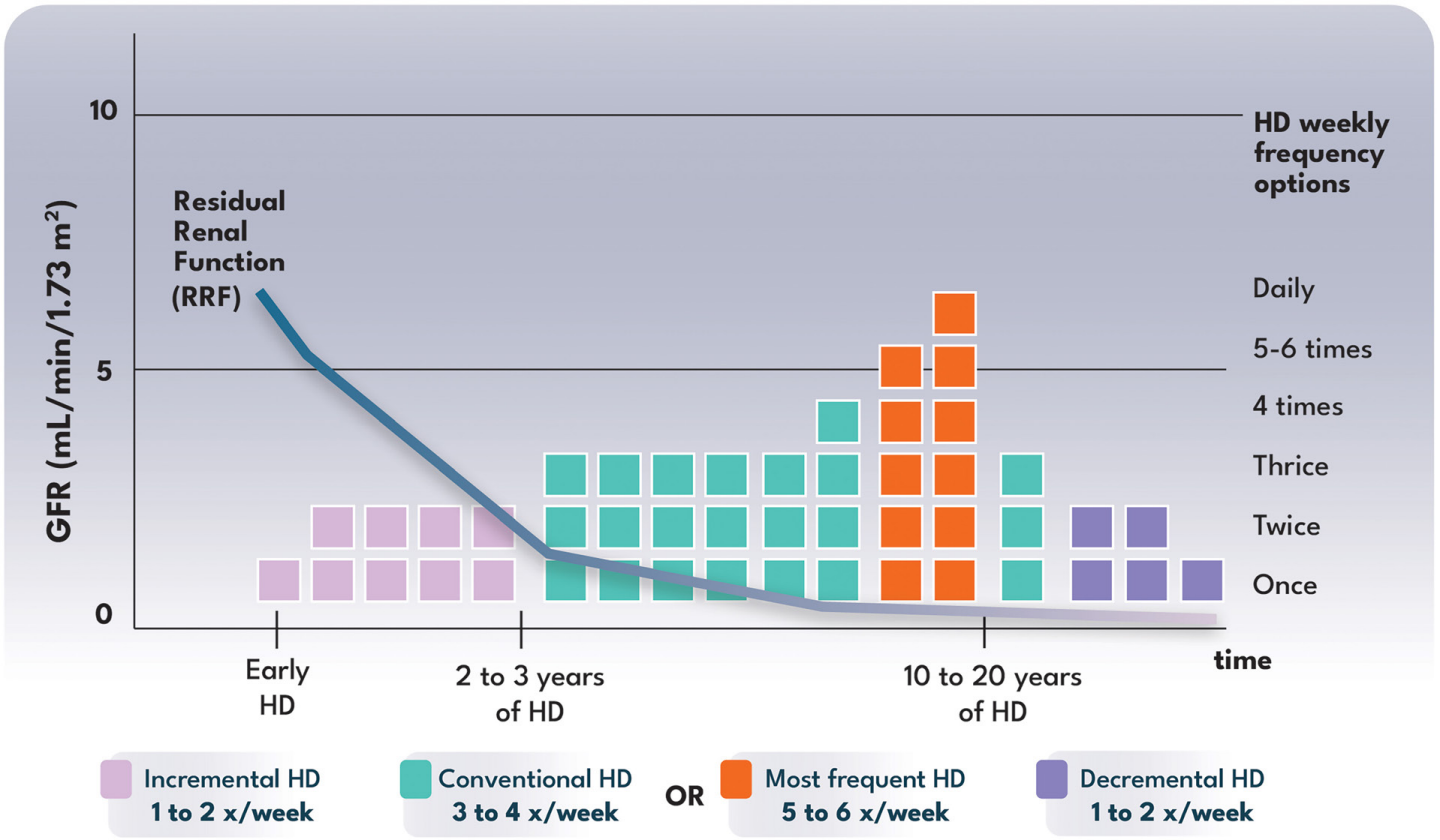
Projection of the need for weekly HD sessions according to residual renal function over time.

Abbreviations: GFR, Glomerular Filtration Rate; HD, Hemodialysis

Source: Adapted from Murea and Kalantar-Zadeh^40^.

Figure 2 presents a schematic model of the patient’s trajectory from incremental PD to the conventional regimen. Figure 3 illustrates the perspective of HD treatment frequency in relation to glomerular filtration rate (GFR) and RRF over time.

### Variations and Examples of the Most Common Incremental Dialysis Regimens

In this section, the main variations and examples of incremental dialysis regimens used in clinical practice are presented. Although different strategies have been proposed, there is still no consensus on the optimal dialysis model in this context.

#### Dialysis Dose Calculation, Residual Renal Function

Since 1998, mathematical models have been developed to measure renal and dialysis clearance^41^. The calculation is based on theoretical models and requires an understanding of the different rates of urea clearance by dialysis and kidneys^42^. Currently, four main methods are described to integrate RRF into dialysis dose calculations. Although rarely used in clinical practice due to their complexity, it is important to understand their fundamentals. The comparison among them is presented in Chart 3. Greater emphasis is placed on methods 1 and 2, which are complementary and most commonly used in clinical practice. In addition to these, there is also a method for estimating RRF based exclusively on laboratory tests (beta-2 microglobulin, creatinine, and urea)^4^.

**Chart 3:** Comparison of methods for calculating and prescribing incremental dialysis

Aspect	Method 1KDOQI Guideline	Method 2Sum of residual and dialysis Kt/V	Method 3Advanced UKM modeling (includes UF)	Method 4SPEEDY
How to calculate	If Kru ≥ 2 mL/min/1.73 m^2^ and clinical/volume stability: 2x/week can be considered; intensify as the RRF falls.	Clearance: Clearance (mL/min) = [[urinary urea × urine volume] ÷ 1440] ÷ mean plasma urea; convert to L/week: mL/min × 10.08; estimate Krt/V and add to the dialytic Kt/V.	Use UKM software to simulate dose/time per session that reaches the target (stdKt/V or EKRUN), incorporating UF and continuous RRF.	Use a spreadsheet/app (SPEEDY) that, based on KRUn and frequency, calculates the Kt/V per session necessary to reach the weekly target.
Advantages	Simple, conservative, easy adoption.	Flexible, customizable, using RRF; methodology familiar to PD/HD.	Theoretically more accurate; includes UF and technical variables.	Practical and clinically oriented; facilitates customization; digital integration (SPEEDY).
Limitations	Restrictive; does not finely quantify renal contribution. Does not apply to PD.	Requires accurate urine collection; urea clearance underestimates GFR (safety margin); does not include UF explicitly.	Complexity, need for software, training, and detailed data. Does not apply to PD.	Requires standardization; maintain close clinical monitoring. Does not apply to PD.
Tools	KDOQI guidelines; simple spreadsheets.	24-hour urine collection; Watson formula for V; touchcalc Kt/V PD calculator.	Solute-Solver and stdKt/V calculators.	SPEEDY spreadsheet (Spreadsheet for the Prescription of incrEmental haEmoDialYsis).

Abbreviations: RRF, residual renal function; Kru, renal urea clearance; KRUn, normalized Kru; UF, ultrafiltration; stdKt/V, weekly standardized Kt/V; Krt/V, residual component of Kt/V; PD, peritoneal dialysis; HD, hemodialysis.

##### • Method 1 (KDOQI)

It suggests using Kru as a monitoring tool. Kru reflects the residual renal clearance capacity and is one of the recommended tools for adjusting the incremental dialysis dose. According to KDOQI guidelines, patients with a Kru of 3 mL/min are equivalent to a weekly Kt/V of approximately 1.0. Therefore, patients with Kru ≥ 2 mL/min/1.73 m^2^ may be eligible for an HD regimen less frequent than three times a week, aiming for a weekly stdKt/V ≥ 2.1. Those on a conventional regimen of three weekly sessions should achieve an spKt/V ≥ 1.2 per session^5^. To calculate Kru, the UpToDate calculator (https://www.uptodate.com/contents/calculator-residual-kidney-urea-clearance-kru-in-hemodialysis-patients) can be used due to its practicality, reliability, and availability, as well as its ability to incorporate variations in the urine collection period. Kru depends on urine volume, the duration of the interdialytic period, and the mean urea concentration (calculated as the sum of the urea levels after the first and second dialysis sessions of the week, divided by two). Because the calculator formula uses BUN (blood urea nitrogen), prior conversion of urea values to BUN is required.

##### • Method 2 (Kt/V)

In this method, the weekly Kt/V derived from RRF is calculated and then added with the weekly Kt/V value for the dialysis sessions. RRF is quantified based on urea clearance, a practice used in both PD and HD. Although urea clearance underestimates true GFR, it provides an adequate safety margin for clinical decision-making.

One way to estimate urea clearance includes^43^:

- Collection of plasma urea before and after two consecutive HD sessions and calculation of the mean value;
- Measurement of 24-hour urine output during the interdialytic interval;
- Measurement of urea concentration in 24-hour urine.

The formula for calculating urinary urea clearance is:

Clearance (mL/min) = [[urinary urea (mg/dL) × urine volume (mL/24h)] × 24 ÷ 1440] ÷ mean plasma urea (mg/dL)

Once urea clearance (Kru) has been calculated, the residual weekly Kt/V (Krt/V) can be estimated, where Kr (L/week) corresponds to urea clearance. Since urea clearance is obtained in mL/min, it must be multiplied by 10.08 in order to convert it to L/week. The time factor (t) is 1 (week), the residual contribution can be expressed as Kru × 10,080 / V, and V can be estimated using the Watson’s formula. The Kr mentioned corresponds to Kru, differing only in the unit of measurement (L/week vs. mL/min).

Another approach is to obtain Krt/V using an online calculator (https://www.zkidney.com/pd-formulas). This calculator was developed for use in PD; therefore, in cases of patients on incremental HD, the “DIALYSATE CLEARANCE” item should be considered to be zero^30,44,45,46^. The standard Kt/V represents the weekly dialysis dose and should be added to the Kt/V derived from weekly residual kidney function to obtain the total weekly Kt/V. A calculator for estimating standard Kt/V is available on the SBN website (https://sbn.org.br/medicos/utilidades/calculadoras-nefrologicas/kt-v/).

Practical example:

Female patient, 40 years old, 74 kg, height 165 cm.

• Urea after the first dialysis session of the week: 38 mg/dL (≈ BUN 17.7 mg/dL)

• Urea before the second dialysis session of the week: 94 mg/dL (≈ BUN 43.9 mg/dL)

• Interdialytic urine volume: 3,550 mL over 44 hours / 2,640 min

• Interdialytic urinary urea: 191 mg/dL

Calculation of KRU using the calculator:

• Urine volume: 3,550 mL

• Urinary urea concentration: 191 mg/dL

• Interdialytic period: 2,640 min

• Initial BUN: 17.7 mg/dL

• Final BUN: 43.9 mg/dL

→ KRU = 7.87 mL/min

Calculation of weekly renal Kt/V:

• K = 7.87 mL/min

• t = 10,080 min (1 week)

• V (Watson) = 33.8 L = 33,800 mL

→ Renal Kt/V = (7.87 × 10,080) / 33,800 = 2.34

Calculation of dialysis Kt/V (weekly), which can be accessed using the SBN calculator:

• Pre-dialysis urea: 94 mg/dL

• Post-dialysis urea: 27 mg/dL

→ spKt/V = 1.37

→ stdKt/V = 1.42

Total weekly Kt/V:

→ Total Kt/V = 2.34 + 1.42 = 3.76

##### • Method 3 (*Urea Kinetic Model* – UKM)

This is considered the most theoretically accurate model, as it incorporates not only continuous clearance by RRF but also the impact of ultrafiltration^42^. It is based on complex mathematical equations that integrate the different forms of solute removal (continuous by the kidneys and intermittent by the machine), in addition to fluid removal.

##### • Method 4 (SPEEDY– *Spreadsheet for the Prescription of incrEmental haEmoDialYsis*)

More recently, this method assumes that the dialysis dose should be proportional to RRF^47^. In this model, the RRF is adjusted to the number of weekly sessions, allowing patients with more preserved renal function to initiate or maintain regimens with lower dialysis frequency. As long as the sum of renal and dialysis clearance reaches the target Kt/V, treatment is considered appropriate. To achieve this, a series of equations are used, in most cases identical to those used by the Solute-Solver, available at the www.ureakinetics.org website. A step-by-step description of the spreadsheet can be found in Casino and Basile^47^.

#### Incremental Dialysis Prescription

The practical models for incremental HD and PD prescriptions are described in Charts 4 and 5.

**Chart 4:** Practical options for incremental hemodialysis^4,8,9,21,31,39^

Strategy	How to apply	Advantages	Risks/cautions
Based on Kru	Kru ≥ 2 mL/min and/or diuresis ≥ 500 mL/24h: **Perform HD 2x/week** Kru ≥ 3.5 mL/min, adherent patient and experienced center: **Perform HD 1x/week in selected cases**.	Preserve RRF.	Subdialysis if overestimated RRF, hypervolemia, high UF rate.
Reduction in the number of sessions	Start 1 to 2x/week when there is substantial RRF, volume stability, and adherence.	Preserves RRF, lower treatment burden.	Subdialysis if overestimated RRF; high interdialytic weight gain.
Reduced session time	2 to 3h per session maintaining 2 to 3x/week.	Less fatigue, easier logistics.	Lower clearance per session, higher UF rate.
Reduced blood flow (Qb) and dialysate flow (Qd)*	Qb 200 to 250 mL/min; Qd 300 to 400 mL/min.	Lower efficiency and fewer symptoms of imbalance.	Reduces Kt/V per session, pay attention to recirculation.
Reduction of HD filter surface area (dialyzer)*	HD dialyzer with smaller surface area.	Reduction of the dialysis dose.	Subdialysis.
Combinations	Combine 2 to 3 measures above with explicit goals.	Fine customization.	Increased monitoring complexity.

Abbreviations: RRF, residual renal function; UF, ultrafiltration; Qb, blood flow; Qd, dialysate flow; HD, hemodialysis.

Note: * The reduction of Qd, Qb, or the surface area of the filter (dialyzer) are complementary measures and should be used with extreme caution, never as a substitute for the minimum dialysis dose or as a practice of cost rationalization (in the case of HD dialyzer and Qd). These strategies are only appropriate when accompanied by measured RRF, eligibility criteria are met, and serial monitoring of adequacy. Their misuse mischaracterizes the incremental approach and contradicts this position.

**Chart 5:** Incremental prescriptions in peritoneal dialysis^34,49,90,91,92^

Modality	Examples of initial prescription	Clinical situations and justifications	Signs of loss of RRF or inadequacy	Recommended progressive adjustments
CAPD (Continuous Ambulatory Peritoneal Dialysis)	– 2–3 exchanges/day of 2 L; – 1 exchange/day of icodextrin (8–16h); – 3 exchanges of 1.5–2 L for 4–6 days/week.	– Patients with diuresis >100 mL/day; – Active lifestyle; – Less exposure to glucose and mechanical overload.	– Increase in urea, creatinine, and phosphorus; – Hypervolemia (edema, weight gain); – Reduction of diuresis; – Uremic symptoms.	– Increase to 3–4 exchanges/day; – Use volumes of 2–2.5 L as per tolerance; – Add nighttime icodextrin; – Move to 7 days/week.
APD (Automated Peritoneal Dialysis)	– Dialysis-free days (3–5 nights/week); – Smaller infusion volume (4–5 night cycles with 1.2–1.5 L); – No dialysis during the day (intermittent night PD; dry day); – Less time connected to the cycler (6–7h sessions, 8–10 L total).	– Greater freedom for work, study, and social activities; – Good RRF to compensate for absence of daytime dwell; – Less time connected to the cycler.	– Reduction in solute removal; – Hypervolemia; – Sharp drop in urine output.	– Increase volume per cycle (up to 2–2.5 L); – Increase number of cycles (up to 5–6); – Change to CCPD; – Expand to 7 nights/week.
Icodextrin (alone or in combination)	– 1 long exchange/day (8–12h).	– Patients with preserved RRF; – Suitable for glucose-free volume control.	– Reduction of diuresis; – Hypervolemia or uremic symptoms.	– Use in long daytime or nighttime exchanges according to the modality, combined with additional exchanges of glucose-based solutions.

Abbreviations: RRF, residual renal function; PD peritoneal dialysis; CCPD, continuous cycling peritoneal dialysis.

### Advantages and Outcomes of Incremental Dialysis Compared with Conventional Dialysis

#### Hemodialysis

In the context of dialysis patient care, RRF is not only a variable used for treatment indication and monitoring, but also an outcome metric to be maintained. The growing body of evidence regarding the maintenance of a clearance and its impact on the survival of patients with CKD-5D is robust, with the publication of NECOSAD data in 2004 initially demonstrating this benefit^25^, an association that has been corroborated over the years^18,26,48^.

When analyzing the impact of incremental HD on the preservation of RRF, some points may be highlighted as symbiotic mechanisms for its occurrence, including (a) greater excretion of sodium and water, leading to (b) better volume control, (c) a lower risk of intradialytic hypotension, and (d) a lower incidence of renal ischemic events^49^. This evidence of maintained renal clearance is observed in cohorts of observational studies, with preservation of urine output, as well as urea and creatinine clearance, in groups that underwent HD less frequently (once or twice a week)^18,50,51,52^.

The use of incremental HD regimens should be carefully considered in the adaptation of patients and their caregivers to the transition of care when starting HD. The time burden, symptoms, and psychological stress of this change should not be minimized, as they have a significant impact on these individuals’ lives. In this context, the TWOPLUS pilot trial, conducted in incident patients with RRF (diuresis ≥ 500 mL/day and GFR ≥ 5 mL/min/1.73 m^2^), compared incremental initiation *versus* conventional HD; patients with incremental onset had a lower burden of psychological symptoms, less fatigue, and a greater sense of autonomy^53^.

Along with clinical benefits, incremental HD has the potential to reduce the environmental burden by decreasing the weekly frequency of sessions. Each HD session at a center is environmentally harmful, with an average of 58.9 kg CO_2_-eq per treatment, in addition to water consumption, with estimates indicating a use of 265 million m^3^/year. Thus, the lower frequency of sessions also reduces weekly water consumption and disposal, relieving pressure on local resources^54,55^. It is important to recognize that there is a lack of studies that directly compare the carbon footprint of incremental *versus* conventional programs^56^. This argument aligns with the call from KDIGO Sustainable Kidney Care^54^ and with ANVISA’s concerns regarding the sustainable use of resources in renal health services.

Regarding clinical outcomes, especially the event of hospitalization in HD patients, evidence from a meta-analysis of 36 studies including 138,939 patients is available. This analysis compared HD regimens performed once or twice a week *versus* HD performed three times a week, in which a lower occurrence of hospitalizations was observed in the incremental group. In the evaluation of potential complications due to the “reduced supply” of dialysis, no statistically significant differences were found between the groups regarding events of hyperkalemia and vascular access dysfunction^24^. Other observational studies suggest a reduction in hospitalizations^48,57^.

In the follow-up of patients on incremental HD, it is important to consider the potential impact of this strategy on survival compared to conventional HD. Meta-analyses show that incremental HD is not associated with a significant increase in mortality compared to conventional HD when applied to appropriately selected patients^15,24^. Similarly, in a non-randomized, multicenter clinical trial involving 132 patients on HD three times a week and 71 patients on HD twice a week, there was no difference in survival between the groups after 24 months of follow-up^58^. In a secondary analysis of the BISTRO trial, a randomized clinical trial, patients with higher GFR at the onset of dialysis therapy continued with longer long-term survival^59^.

#### Hemodiafiltration (HDF)

The evidence regarding the use of HDF in an incremental modality presents mechanistic plausibility due to its greater removal of medium-sized molecules when the appropriate convective dose is reached during the session^60^. A case series including 11 incident dialysis patients evaluated the use of HDF once a week during follow-up, with an escalation of therapy to two to three times a week over a median period of 7 months (IQR 3–24 months)^61^. However, as of the publication of this statement, we still lack evidence comparing its use and benefits with those of conventional HDF prescription or even HD as controls.

#### Peritoneal Dialysis

Initial reports on incremental PD focused on demonstrating the feasibility of the technique and its clinical safety, using targets based on Kt/V. Burkart and Satko, in 2000, described a small group of 13 CAPD patients followed for two years, confirming the possibility of maintaining incremental treatment with adequate clearance parameters^62^. In the same year, De Vecchi et al.^63^ followed 25 patients initially treated with one to two daily exchanges, observing a low rate of peritonitis and greater acceptance of the reduced exchange regimen when compared to traditional regimens of three to four exchanges.

Although initially conceived to rely on RRF to enable prescription, later evidence suggests that the incremental approach not only benefits from RRF, but may also contribute to its preservation, including maintenance of diuresis, clearance, and a slower decline over time^64,65,66,67,68,69,70,71^.

The survival of the technique appears as a parameter favorably associated with incremental PD. Fernandes et al.^70^ demonstrated greater technical survival in patients treated incrementally. Convergent results were identified by Sakurada et al.^72^, in an analysis of a registry of adult patients incident on PD between 2007 and 2017, revealing a lower need for early transfer to HD in the first month of therapy. A potential explanatory mechanism lies in the lower exposure to bioincompatible peritoneal solutions, due to the lower number of exchanges and lower cumulative load of glucose, osmolarity, and acidity.

Improvement in quality of life is one of the central objectives of incremental PD, due to the fewer procedures and lower therapeutic burden. Naljayan et al.^73^, in a retrospective North American study with CAPD patients, identified better scores on the Kidney Disease Quality of Life (KDQOL) questionnaire, particularly in the domains of physical component, burden of kidney disease, and effects of kidney disease. Regarding the procedural load, the lower number of connections and disconnections suggests an operational benefit.

From an infectious safety perspective, four studies reported a lower risk of peritonitis associated with incremental PD^64,66,72,74^. However, more recent findings from a large multicenter cohort of the PDOPPS, which followed incident patients between 2014 and 2017 in incremental or conventional modality, did not demonstrate significant differences in this outcome^75^.

Studies evaluating hospitalization^66,70,74,76^ showed an association between incremental PD and lower hospitalization rates, including among patients with *diabetes mellitus* (DM)^74^. These findings reinforce the potential of the incremental approach to reduce hospital complications and interactions, possibly related to the maintenance of RRF, reduced exposure to bioincompatible solutions, and a better therapeutic experience.

The results regarding patient survival are encouraging. Lee^28^, comparing 232 patients in conventional and 71 in incremental PD, observed similar survival between the groups, with a significant advantage among patients with DM treated incrementally. Liu et al.^69^, evaluating 285 incident patients in incremental CAPD *versus* 502 in full CAPD, demonstrated a 39% reduction in the risk of death from all causes and a 41% reduction in the risk of mortality in the first six years. Yan et al.^74^, in a Canadian retrospective cohort with 175 patients, reported low peritonitis and hospitalization, although a higher initial infusion volume was associated with higher mortality. Fernandes et al.^70^, in a Portuguese cohort, also reported higher survival associated with the incremental technique. Finally, results from PDOPPS centers showed no difference in patient mortality^75^.

Despite the growing body of observational evidence, only one randomized controlled trial has been identified. Yan et al.^77^ compared 70 patients on CAPD undergoing three daily exchanges *versus* 69 patients undergoing four exchanges, followed for 24 months, with no differences between the groups in patient or technique survival^28^. In the field of systematic reviews, Garofalo et al.^15^ analyzed 22 studies of incremental PD and HD, seven of which were specifically on incremental PD, finding no significant differences in RRF, patient survival, technique survival, or time to escalation to full dose after 24 months. Xu et al.^28^, evaluating ten publications, confirmed comparable outcomes between incremental PD and conventional PD in incident patients, with no differences in mortality, peritonitis, or technical survival.

In summary, the body of available literature, although mostly composed of observational studies, points to the feasibility and potential efficacy of incremental PD, suggesting benefits related to the maintenance of RRF, lower hospitalization rates, and possible improvement in quality of life, without consistent evidence of increased risk when compared to conventional PD.

### Challenges, Organization, and Legal Aspects

Although conceptually simple, the implementation of incremental dialysis involves a number of organizational, logistical, and regulatory challenges. Because it is an individualized and dynamic prescription model that depends on the systematic monitoring of RRF and the progressive adaptation of the dialysis dose, its application requires integration within a multidisciplinary team, well-structured protocols, and institutional support. In addition, legal and administrative aspects become relevant, since incremental dialysis is not yet widely recognized in some health systems as a specific modality of renal replacement therapy. This may generate impasses related to authorization, reimbursement, and monitoring. Discussing these elements is essential to enable the safe and sustainable expansion of incremental dialysis and to ensure equity of access to this promising dialysis initiation strategy.

#### Risks of Subdialysis and Resistance to Transition

Clinical risks of subdialysis include volume overload, hyperphosphatemia, hyperkalemia, and metabolic acidosis. The mitigation of these risks depends on rigorous monitoring through serial laboratory tests and systematic clinical evaluation with monitoring of urine volume. In addition, high ultrafiltration rates in spaced sessions pose an additional concern, especially in a once-weekly regimen^78^.

The literature on patient resistance to the transition from incremental dialysis to the conventional regimen remains scarce. Recent implementation protocols incorporate measures of acceptability and patient-reported outcomes^79^, recognizing that the subjective perception of well-being can interfere with adherence to dialysis dose escalation. To deal with this resistance, it is recommended to define objective criteria for therapy intensification in advance, as exemplified in Chart 2. Periodic review of the therapeutic plan, combined with the integration of support strategies – including nutritional guidance, volume control, and potassium and phosphorus management – favors a safer and less traumatic transition for the patient^78,79^.

#### Organization of the Dialysis Center and Role of the Multidisciplinary Team

The implementation of incremental dialysis requires an adapted organizational structure to ensure safety, effectiveness, and optimization of dialysis slots. The experience of centers that have adopted this model demonstrates that strategic planning and standardization of protocols are fundamental for the sustainability of the practice. A practical example of how HD centers can organize themselves to optimize capacity and resources is presented in Chart 6.

**Chart 6:** Suggested organization of hemodialysis centers to optimize incremental dialysis slots (1–2 sessions/week)

Monday	Tuesday	Wednesday	Thursday	Friday	Saturday
Patient 1 2x/week	Patient 2 2x/week	Patient 3 2x/week	Patient 1 2x/week	Patient 2 2x/week	Patient 3 2x/week
Patient 4 2x/week	Patient 5 2x/week	Patient 6 1x/week	Patient 7 1x/week	Patient 4 2x/week	Patient 5 2x/week

From the time of admission, it is essential that the center has a predefined escalation plan with objective criteria for therapy progression (Chart 2). The care schedule should be planned in a flexible way, providing for sessions at reduced frequency (one to two times a week) and enabling a rapid transition to three times a week as necessary^40,80,81^.

The care routine should include continuous and systematized monitoring, including recording of diuresis, serial measurement of laboratory tests at predefined intervals, periodic evaluation of RRF, and auditing of adverse events such as hospitalizations^40,82^, according to Chart 1.

It is recommended that clinical performance indicator panels be adopted, which include not only objective parameters—such as RRF decline, laboratory tests, and volume overload—but also patient-centered outcomes, such as fatigue, pruritus, and individual preferences, to assess the need for unscheduled escalation^79,81,83^.

The effectiveness of the strategy depends on an integrated multiprofessional team, with well-defined attributions among the nephrologist^80^, the nursing staff^82^, nutritionists^84^, clinical pharmacy^79^, and the social work and psychology teams^85,86^ regarding: (a) patient and family education; (b) nutritional and dietary monitoring; (c) monitoring of urine output and clinical and laboratory parameters; and (d) standardization and updating of institutional protocols. It is worth noting that structured pre-dialysis education programs, with the active participation of the multidisciplinary team, can improve the transition of care and favor more assertive, informed choices^84^.

#### Legal, Ethical, and Financing Aspects

Although the main guidelines recognize incremental dialysis as a valid and important dialysis initiation strategy, detailed guidance on its practical application is still limited, contributing to heterogeneity across countries and dialysis centers^4^. In Brazil, dialysis is predominantly funded by the Unified Health System (SUS), and modalities other than conventional dialysis are generally restricted to patients with private health insurance. Personalization through incremental dialysis may reduce costs by decreasing the consumption of water, supplies, and care staff^4^. Estimates suggest that, if widely implemented in the United States, Medicare could save between $250 million and $500 million annually^87^. Currently, in Brazil, there is no specific remuneration for incremental dialysis, nor are there incentives for more sustainable and/or patient-centered dialysis.

From an ethical standpoint, studies have reported the safety and acceptability of the incremental approach^12^. The basis of this strategy is shared decision-making, involving physicians, multidisciplinary teams, patients, and family members. This approach presupposes co-responsibility in symptom monitoring, treatment adherence, and continuous review of therapeutic goals. The benefits and risks of incremental dialysis should be evaluated individually, ensuring that the dialysis prescription respects not only clinical criteria, but also the values, preferences, and quality of life of each patient^88^.

Incremental dialysis is a viable, safe, and personalized alternative for patients with preserved RRF. Its adoption, however, still faces regulatory, legal, and funding barriers. The consolidation of this practice requires updating national guidelines, revising cost models, strengthening the role of multiprofessional teams, and the active participation of patients in therapeutic decisions.

### Patient Perspectives

End-stage chronic kidney disease (CKD-5D) poses considerable challenges to patients’ lives, which often extend far beyond clinical limitations. Conventional dialysis treatment, with its rigid and intensive routine, may represent a new burden on individuals who have already been living with severe restrictions during the conservative phase of the disease. In this context, the pre-dialysis experience is usually marked by strict dietary and fluid restrictions, fear of decompensation, and a feeling of loss of autonomy. Many patients describe this period as a controlled existence, permeated by the anxiety of “making mistakes” and overloading the kidneys. Paradoxically, the initiation of dialysis comes to be seen not only as a medical necessity but as a possible sense of liberation from previous restrictions. It is precisely at this point that incremental dialysis emerges as a transformative alternative. Unlike the rigid model of immediate initiation at full dose, incremental dialysis proposes a more humane transition, which respects the patient’s RRF and gradually adjusts the treatment^14,17,34,70,89^. This approach promotes a less traumatic adaptation, reduces physical and emotional burden, and restores to the patient some of the lost freedom and quality of life.

Traditionally, upon arrival at dialysis, the patient’s path is usually the direct initiation of a full-dose regimen – three weekly HD sessions or the equivalent in PD. However, many individuals still have RRF, capable of contributing to metabolic balance. Incremental dialysis values this potential, gently complementing what the body can still accomplish, and gradually increasing the dose as RRF decreases^17,34^.

From the patient’s standpoint, the main benefits are felt in different dimensions:

- Fewer restrictions, more quality of life: by preserving RRF, incremental dialysis allows for greater nutritional and fluid flexibility. This means eating with less fear, drinking with less guilt, and living more autonomously. For patients who have spent months or years under a restrictive conservative regimen, this is an immeasurable benefit.
- Gradual adaptation to dialysis: the start of dialysis is a difficult emotional milestone. The possibility of starting with a lighter regimen makes adaptation less traumatic, reducing the feeling of loss of control and increasing psychological resilience^31^.
- Free time and autonomy: instead of being subjected from the beginning to an exhaustive schedule of sessions, the patient retains more space in their routine. This preserves not only their social and professional life but also the feeling that the disease has not completely taken over their daily life.
- Preparing for the future: in cases where disease progression makes the need for full dialysis inevitable, incremental dialysis already works as a training period. The patient gradually adapts to the new reality, without sudden ruptures, which improves adherence and clinical outcomes.

Incremental dialysis transcends the technical aspect of dialysis prescription: it represents a paradigm shift, by recognizing the patient as the protagonist of their own treatment. By integrating science and empathy, this approach preserves RRF, reduces the therapeutic burden, favors nutritional and metabolic balance, improves psychological well-being, and promotes a gentler transition to full dialysis.

More than prolonging life, it offers conditions for this life to be lived more fully.

### SBN Recommendations on How to Perform Incremental Dialysis

**Figure 4:**
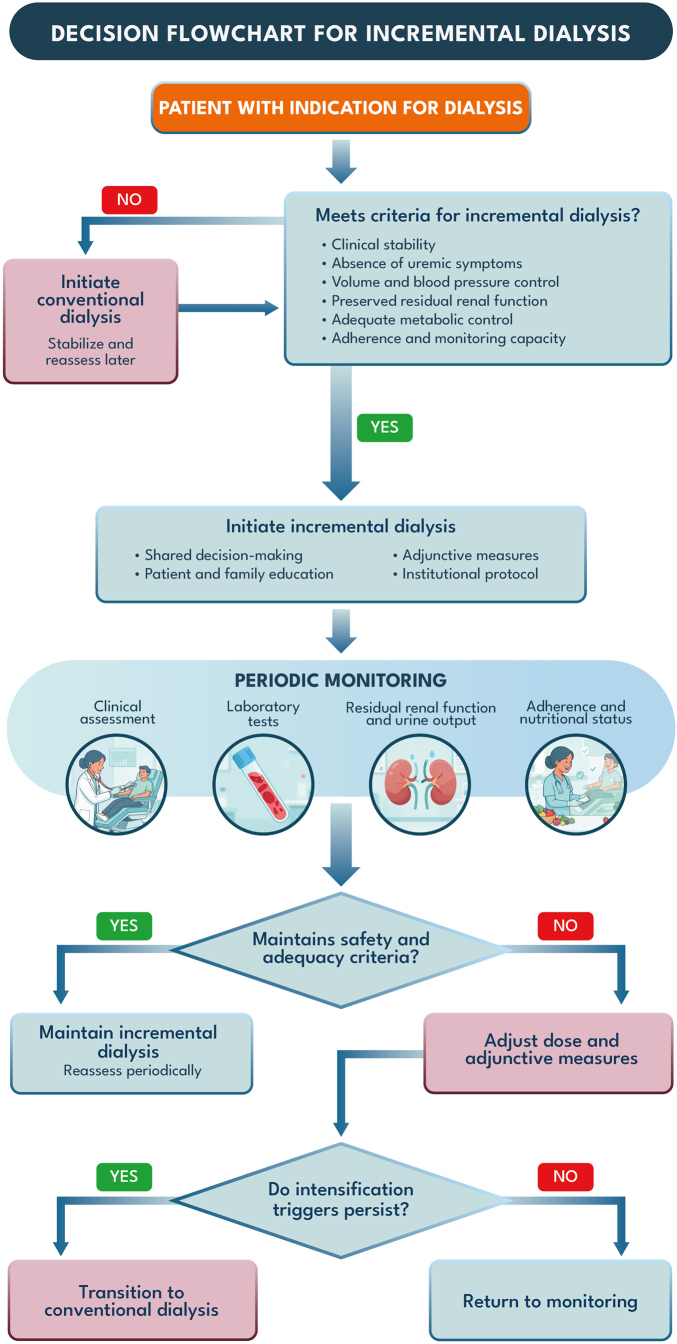
Decision flowchart for incremental dialysis.

Based on the scientific evidence and expert opinion presented throughout this position, the SBN recommends the implementation of incremental HD and PD, as summarized in Chart 7. To facilitate the practical application of the recommendations, we have included a decision-making flowchart (Figure 4) that objectively summarizes the steps for implementing incremental dialysis.

**Chart 7:** SBN recommendations and guidance on how to perform incremental dialysis

Recommendations	How to do it
Implementation	Carry out an institutional protocol according to the local reality.
Trained multiprofessional team	Train physicians, nurses, nutritionists, psychologists, and social workers on incremental dialysis to assist in successful therapy.
Evaluate inclusion criteria for patients on incremental dialysis	HD ≥ 500 mL of urine output; PD ≥ 100 mL of urine output; Kru ≥ 2 mL/min/1.73 m^2^; Absence of hypervolemia and uncontrolled arterial hypertension; Absence of uremic symptoms; Adequate control of mineral and bone metabolism; Adequate potassium control (potassium ≤ 5.0 mEq/L); Adequate control of volume status (interdialytic weight gain < 5% of dry weight); Patient adherence to treatment and monitoring capacity; Infrequent hospitalization not related to dialysis prescription; Absence of persistent anemia that is difficult to control; Good nutritional status, without marked hypercatabolic state.
Shared decision	Shared decision-making with the patient, family members, and the medical and multidisciplinary team.
Patient and family education about incremental therapy	Advise the patient on the need for urine monitoring, testing, diet, medications, and the need to transition to conventional therapy when indicated.
Prescribe medications that increase RRF and help control incremental dialysis	Diuretics, especially loop diuretics; Oral sodium bicarbonate; Phosphorus binders; Potassium binders.
Diet guidance by a nutritionist	Low-sodium diet; Potassium and phosphorus restriction; Adequate protein intake.
Patient monitoring on incremental dialysis	The parameters that need to be monitored are: – Laboratory tests (blood count, potassium, venous blood gases, phosphorus, parathyroid hormone); – Clinical evaluation in all sessions and consultations (hypervolemia, uremia); – Residual urine output; See details and frequency in Chart 1.
Criteria for dialysis intensification	**Clinical:** Signs of hypervolemia, symptoms attributable to uremia, malnutrition, anuria, hospitalization for hypervolemia, or uremia. **Laboratory:** Refractory anemia, bone mineral metabolism disorders, hyperkalemia, or refractory metabolic acidosis. See details in Chart 2.
Systematic registration and documentation	Document the initiation of therapy and the criteria, as well as clinical and laboratory evolution and adjustments, with justifications on a monthly basis.
Clinical outcome assessment	Assess outcomes such as hospitalization, complications, residual urine output, and transition to conventional therapy.

Abbreviations: HD, hemodialysis; PD, peritoneal dialysis. Note: Although there are randomized controlled trials and meta-analyses suggesting similar survival between incremental dialysis and conventional dialysis, the recommendations of this SBN Guideline are based on the currently available evidence—predominantly observational—and expert consensus, with a focus on standardization, safety, and applicability to the Brazilian context.

## Conclusion

Incremental dialysis represents a rational, safe, and evidence-based strategy for initiating dialysis therapy. Studies have shown that it contributes to the preservation of RRF, reduces exposure to risks inherent in conventional dialysis, and improves quality of life. When applied to carefully selected patients and accompanied by appropriate monitoring, the incremental approach is not associated with higher mortality or increased complications.

In addition to the clinical benefits, the strategy contributes to the sustainability of the health system by optimizing the use of resources without compromising the effectiveness of the treatment. It is a strategy that favors an individualized, patient-centered care model—values that are aligned with the principles of contemporary care.

Given this scenario, the SBN reaffirms that incremental dialysis is a valid, safe, and sustainable alternative, which should be incorporated in a structured manner into dialysis centers, with clear criteria for eligibility, monitoring, and therapeutic transition.

## Data Availability

No additional data is available
