# Supplementary material for: Position Statement of the Brazilian Society of Nephrology on incremental dialysis
Source: J Bras Nefrol. 2026 Jun 26;48(3):e20250374. doi: 10.1590/2175-8239-JBN-2025-0374en (PMC13309009; doi:10.1590/2175-8239-JBN-2025-0374en)
Supplement: Translation

# Translation: Posicionamento da Sociedade Brasileira de Nefrologia sobre diálise incremental

**DOI:** 10.1590/2175-8239-JBN-2025-0374pt

**Authors:** Fernanda Salomão Gorayeb-Polacchini, Thyago Proença de Moraes, Rosilene Motta Elias, Allison Andrade, Dirceu Reis da Silva, Felipe Costa Neves, Fernando das Mercês de Lucas, Isadora Cartaxo de Sousa Calvo, Juliana El Ghoz Leme, Maria Gabriela Guimarães, Pasqual Barretti, Patrícia Ferreira Abreu, Paulo Henrique Santos Fraxino, Ramon Lima, Stenio Barbosa de Freitas, Viviane Calice-Silva, José Andrade Moura-Neto

**Affiliations:** 1 Sociedade Brasileira de Nefrologia, São Paulo, SP, Brasil.; 2 Hospital de Base, FUNFARME, São José do Rio Preto, SP, Brasil.; 3 Pontifícia Universidade Católica do Paraná, Curitiba, PR, Brasil.; 4 Nefroclínicas Curitiba, PR, Brasil.; 5 Universidade de São Paulo, São Paulo, SP, Brasil.; 6 Universidade Nove de Julho, São Paulo, SP, Brasil.; 7 Hospital de Clínicas de Porto Alegre, Porto Alegre, RS, Brasil.; 8 Instituto de Doenças Renais, Porto Alegre, RS, Brasil.; 9 UNISINOS, São Leopoldo, RS, Brasil.; 10 Escola Bahiana de Medicina e Saúde Pública, Salvador, BA, Brasil.; 11 Hospital Ana Nery, Departamento de Nefrologia, Salvador, BA, Brasil.; 12 Universidade Federal da Bahia, Programa de Pós-Graduação em Ciências da Saúde, Salvador, BA, Brasil.; 13 International Renal Research Institute of Vicenza, Vicenza, Italia.; 14 Universidade Estadual Paulista, Faculdade de Medicina de Botucatu, Botucatu, São Paulo, Brasil.; 15 Universidade Federal de São Paulo, São Paulo, SP, Brasil.; 16 Hospital São João de Deus, Divinópolis, MG, Brasil.; 17 Fundação Pró-rim, Joinville, SC, Brasil.; 18 Universidade da Região de Joinville, Joinville, SC, Brasil.

**Keywords:** Diálise Renal, Hemodiálise, Diálise Peritoneal, Falência Renal Crônica, Terapia Renal Substitutiva, Qualidade de Vida, Diálise Incremental, Consenso

## Abstract

A diálise incremental, aplicável tanto à hemodiálise (HD) quanto à diálise peritoneal (DP), é uma abordagem individualizada que consiste em oferecer a dose de diálise ajustada à função renal residual (FRR) do paciente, de modo a alcançar os mesmos desfechos clínicos observados com doses plenas, porém, oferecendo melhor qualidade de vida e menor exposição aos riscos associados ao procedimento dialítico. Este Posicionamento da Sociedade Brasileira de Nefrologia (SBN) tem como objetivo apresentar recomendações sobre critérios de elegibilidade, prescrição, monitorização e implementação segura, bem como relatar os resultados clínicos já descritos com essa abordagem. A elegibilidade inclui uma diurese residual ≥ 500 mL/24h na HD ou ≥ 100 mL/24h na DP e/ou um *clearance* de ureia (Kru) ≥ 2,0 mL/min/1,73 m^2^, além de estabilidade clínica e controle volêmico e metabólico. Recomenda-se a monitorização com reavaliação regular da FRR. Os indicadores para intensificação da dose dialítica incluem hipervolemia, sintomas urêmicos, piora do estado nutricional, hiperpotassemia, hiperfosfatemia e acidose metabólica refratária, além de quadro laboratorial de subdiálise. A implementação da diálise incremental demanda protocolos institucionais bem definidos, educação sistemática de pacientes e familiares, uma equipe multiprofissional devidamente capacitada e um processo de decisão compartilhada. As evidências científicas sugerem que a diálise incremental é segura e efetiva, atenuando a perda da FRR, podendo reduzir hospitalizações, mantendo ou melhorando a qualidade de vida, sem aumentar a mortalidade. Adicionalmente, pode contribuir para a redução de custos e para a maior sustentabilidade do sistema de saúde, devendo ser considerada parte integrante do arsenal terapêutico contemporâneo.

## Introdução

A prescrição da diálise com base na cinética da ureia (Kt/V) foi estabelecida na década de 1980 e consolidou-se como padrão em pacientes com doença renal crônica em diálise (DRC-5D)^1,2^. Tradicionalmente, esse modelo define uma prescrição uniforme, centrada na frequência e na duração das sessões, sem considerar as variações individuais da função renal residual (FRR).

Em 1985, Bonomini e colaboradores introduziram o conceito de diálise incremental, propondo ajustar a intensidade do tratamento conforme a FRR^3^. Essa abordagem reconhece que, no início da terapia dialítica, entre 30% e 70% dos pacientes mantêm FRR significativa, cuja contribuição para a depuração global é frequentemente subestimada^4,5^. A FRR é definida pela presença de diurese de 24 horas ≥ 100 mL ou por um *clearance* de ureia (Kru) ≥ 2,0 mL/min/1,73 m^2 5,6^. Sua manutenção é clinicamente relevante, uma vez que contribui para a depuração de toxinas urêmicas de maior peso molecular e para a manutenção da homeostase hídrica, eletrolítica e metabólica^6,7^.

A hemodiálise (HD) convencional é definida como a realização de três a quatro sessões semanais, com duração de três a cinco horas cada. Na prática, prevalece o esquema de três sessões por semana, com quatro horas de duração, regime que foi historicamente estabelecido e que demonstrou eficácia em melhorar a sobrevida e proporcionar estabilidade clínica e metabólica. No entanto, iniciar o tratamento dialítico com dose plena em pacientes com FRR preservada pode representar uma exposição desnecessária aos riscos inerentes à terapia e às suas complicações, além de potencialmente acelerar a perda da FRR. As diretrizes do *Kidney Disease Outcomes Quality Initiative* (KDOQI) recomendam que pacientes em HD com Kru < 2,0 mL/min/1,73 m^2^ recebam, no mínimo, três sessões por semana, com pelo menos três horas por sessão^5^. Embora vise garantir uma adequação dialítica mínima, essa recomendação não reflete, obrigatoriamente, as necessidades individuais de pacientes com FRR preservada.

Atualmente, a adequação dialítica não deve ser definida apenas pelo Kt/V. Deve incorporar indicadores complementares, como FRR, condições clínicas e laboratoriais, qualidade de vida e preferências do paciente. O modelo incremental integra a depuração renal residual à depuração dialítica para compor a depuração total, buscando um Kt/V semanal *(Kt/V standard-stdKt/V)* alvo, compatível com a adequação clínica e metabólica. Dessa forma, a diálise incremental substitui o paradigma de prescrição uniforme por uma abordagem individualizada, dinâmica e centrada no paciente, com uma transição gradual para o regime convencional à medida que ocorre a perda da FRR^5,8,9^.

Na prática, muitos pacientes iniciam a diálise em contexto de urgência, frequentemente com uremia, distúrbios hidroeletrolíticos, sobrecarga volêmica, anemia e alterações do metabolismo mineral e ósseo. Nesses casos, é adequado iniciar com dose plena de diálise, visando à estabilização clínica e à correção das alterações metabólicas. Após o controle do quadro e a adequada avaliação da FRR, pode-se considerar a redução progressiva da intensidade do tratamento, desde que sejam mantidos critérios rigorosos de segurança e monitorização. É essencial explicitar essa abordagem dinâmica para evitar uma interpretação equivocada de que a diálise incremental deve ser adotada de forma imediata e universal, reforçando seu caráter individualizado e dependente do contexto clínico.

Na DP, o conceito da abordagem incremental encontra-se mais consolidado, com conceituação e recomendação desde 1997^10^. A *International Society for Peritoneal Dialysis* (ISPD) recomenda, desde 2006, o início da terapia com regimes parciais de DP, aumentando gradualmente o número de trocas à medida que ocorre o declínio da FRR^11^. Evidências demonstram que a abordagem incremental em DP apresenta mortalidade semelhante à dos regimes convencionais, com maior preservação da FRR, melhor qualidade de vida, menor exposição a soluções bioincompatíveis, menor risco de peritonite e redução de insumos e custos relacionados ao tratamento^12,13^.

Na HD, a abordagem incremental tem recebido crescente atenção nos últimos anos. Estudos comprovam que a estratégia é segura e eficaz, associando-se à melhor preservação da FRR, níveis mais baixos de inflamação e redução de hospitalizações e custos assistenciais^14,15,16,17,18,19,20^, com mortalidade similar à observada na HD convencional, desde que os critérios de elegibilidade e monitoramento sejam instituídos^17,20,21,22^.

A relevância clínica da preservação da FRR é amplamente documentada. Evidências sugerem que iniciar a diálise em regimes convencionais ou intensivos pode acelerar a perda da FRR, possivelmente em razão de episódios recorrentes de hipotensão intradialítica, flutuações hemodinâmicas e ativação inflamatória^23,24^. Pacientes com FRR preservada apresentam menor risco cardiovascular, melhor estado nutricional, maior controle da anemia e da sobrecarga volêmica, além de melhor qualidade de vida^24,25,26^. Assim, independentemente da modalidade de diálise, devem ser adotadas estratégias para preservar a FRR, incluindo controle pressórico e glicêmico adequados e evitando o uso de drogas nefrotóxicas.

No Brasil, o Censo de Diálise da Sociedade Brasileira de Nefrologia (SBN) de 2024 registra mais de 170 mil indivíduos em terapia dialítica; entretanto, apesar do número expressivo, desconhece-se a proporção de pacientes sob regime incremental^27^. Diante da magnitude da população em diálise, da heterogeneidade clínica dos pacientes e da necessidade de otimização de recursos no Sistema Único de Saúde (SUS), um posicionamento oficial da SBN sobre a diálise incremental é fundamental. Esse posicionamento busca estabelecer critérios de elegibilidade, parâmetros de monitoramento e orientações práticas para a implementação segura da estratégia, permitindo avanços tanto na personalização do cuidado quanto na sustentabilidade do sistema de saúde.

## Métodos

O presente documento constitui um posicionamento oficial da SBN, elaborado por um grupo técnico composto por membros da Diretoria, do Departamento de Diálise, do Comitê de Diálise Peritoneal e do Comitê de Pacientes. O processo de elaboração incluiu uma revisão narrativa e a síntese crítica da literatura científica.

A redação das recomendações seguiu deliberação por consenso clínico, e a versão final do documento foi revisada e aprovada por todos os autores. Trata-se, portanto, de um posicionamento institucional de caráter educativo, não normativo, cujo objetivo é orientar a prática clínica e promover a uniformização de condutas em torno da diálise incremental no contexto brasileiro.

## Discussão

A diálise incremental representa uma estratégia individualizada de prescrição dialítica, fundamentada no aproveitamento da FRR do paciente. Diferentemente dos esquemas convencionais, o regime incremental propõe ajustes progressivos e personalizados que permitem alcançar os mesmos resultados clínicos e metabólicos obtidos com regimes plenos, porém com menor exposição à diálise^28^.

### Indicações da Diálise Incremental

A aplicação da diálise incremental requer critérios clínicos e laboratoriais bem definidos, além de monitoramento rigoroso de parâmetros de segurança para reduzir o risco de eventos adversos^11,29^. Os principais critérios incluem:

- **Função renal residual**: para pacientes em DP, é definida, de maneira prática, como um volume urinário superior a 100 mL/24h^11,29^. No caso da HD, recomenda-se a prescrição incremental quando houver pelo menos 500 mL de débito urinário em 24 horas^11,29,30^.

Quando a coleta de urina de 24 horas não for possível, a FRR pode ser avaliada por métodos distintos de mensuração. O primeiro é o **registro domiciliar da diurese**, no qual o paciente mede e anota o volume urinário produzido em 24 horas, utilizando um recipiente graduado e seguindo orientação padronizada da equipe. Esse método, apesar de simples, oferece uma estimativa prática e relativamente confiável da FRR.

Uma alternativa é a **dosagem sérica de beta-2 microglobulina**. Valores persistentemente elevados (acima de 30 mg/L)—desde que na ausência de infecções, processos inflamatórios agudos ou condições que elevem o marcador independentemente da função renal—indicam declínio significativo da FRR. Nessas situações, a estratégia incremental deixa de ser apropriada, devendo-se considerar o ajuste para um regime dialítico convencional^4^.

- **Sintomatologia**: ausência de sinais e/ou sintomas associados à uremia.
- **Controle adequado do estado volêmico**: o paciente deve manter estabilidade volêmica, sem episódios recentes de hipervolemia ou descontrole pressórico atribuídos à prescrição da diálise. Em HD, o ganho interdialítico de peso não deve ultrapassar 5% do peso corporal seco estimado entre as sessões^31^.
- **Adesão ao tratamento e capacidade de monitoramento**: pacientes motivados, com suporte familiar ou social e capazes de compreender e seguir o esquema de diálise adaptado, bem como as orientações de dieta e medicações. É fundamental que, desde o início, sejam orientados de que se trata de uma estratégia transitória, sujeita a ajustes mensais conforme avaliação clínica.
- **Estabilidade clínica e ausência de internações recentes:** o paciente deve apresentar estabilidade clínica, sem internações recentes decorrentes de descompensação volêmica, sintomas urêmicos, infecção grave ou intercorrências metabólicas; por outro lado, hospitalizações ocasionais, que não sejam associadas à necessidade de ajuste imediato da dose dialítica, não contraindicam a estratégia incremental. A frequência e o motivo das internações devem ser considerados parâmetros importantes na avaliação periódica da elegibilidade e segurança para manutenção do regime incremental.
- **Controle da anemia**: o paciente deve apresentar hemoglobina estável, sem sinais de gravidade ou refratariedade, com resposta adequada ao tratamento (agentes estimuladores da eritropoiese, suplementação de ferro e correção de deficiências). A persistência de anemia moderada a grave ou a ausência de resposta, apesar de manejo otimizado, deve levar à reavaliação da prescrição, da elegibilidade para diálise incremental e à investigação de causas secundárias^11,29^.
- **Bom estado nutricional:** o paciente deve apresentar estado nutricional adequado, sem evidências de hipercatabolismo ou perda proteico-energética significativa. Deve haver estabilidade dos parâmetros laboratoriais nutricionais, bem como ingestão alimentar compatível com as recomendações dietéticas. A presença de desnutrição, inflamação ou catabolismo acentuado deve motivar intervenção nutricional específica e reavaliação da elegibilidade para a diálise incremental^11,29^.
- **Controle adequado do potássio:** o paciente deve apresentar níveis séricos de potássio dentro da faixa de normalidade, sem episódios recentes de hiperpotassemia clinicamente significativos. Potássio sérico ≤ 5,0 mEq/L é considerado um parâmetro seguro para manutenção da estratégia incremental. Alterações transitórias podem ocorrer, mas requerem avaliação da adesão dietética, revisão da prescrição de diálise e ajuste de medicamentos que interferem na homeostase do potássio. Em casos de hiperpotassemia recorrente ou refratária, deve-se considerar a intensificação do regime dialítico. Na ausência de outros sintomas associados à uremia, estratégias medicamentosas para controle do potássio podem ser empregadas em conjunto com a estratégia incremental^11,29^.
- **Controle adequado do metabolismo mineral e ósseo (DMO-DRC):** o paciente deve apresentar níveis séricos de paratormônio (PTH), cálcio e fósforo dentro das faixas recomendadas^11,29^.

### Monitoramento da Adequação Dialítica e Ajustes da Terapia

O monitoramento na diálise incremental é contínuo e exige avaliação periódica do paciente. Esse monitoramento deve ser feito com base em parâmetros clínicos e laboratoriais. A periodicidade de cada parâmetro encontra-se descrita no Quadro 1 ^11,32,33,34^.

**Quadro 1:** Proposta de monitoramento mínimo do paciente em diálise incremental

Avaliação clínico-laboratorial	Periodicidade de acompanhamento		
	Semanal	Mensal	Trimestral
Pressão arterial pré-diálise e hipotensão intradialítica (pacientes em hemodiálise)	X		
Avaliação clínica do estado nutricional		X	
Fragilidade e índice de desempenho de *Karnofsky*			X
Qualidade de vida e expectativa de vida			X
Avaliação psicológica		X	
Diurese residual		X	
Função renal residual/*clearence* de ureia			X
Controle do potássio e bicarbonato		X	
Controle de cálcio e fósforo		X	
Controle do paratormônio e perfil de ferro			X
Controle da anemia		X	

Fonte: Adaptado de Tangvoraphonkchai e Davenport, 2019.

- **Exames laboratoriais de rotina**: ureia, creatinina, potássio, gasometria venosa, cálcio, fósforo, PTH, hemoglobina, estoque de ferro e albumina sérica.
  - Em DP, o Kt/V total ≥ 1,7 semanal (peritoneal + renal) deve ser medido, mas não é indicado de maneira isolada para definir a dose da diálise, sendo fundamental que o paciente mantenha o bem-estar, o controle clínico e laboratorial, a nutrição adequada e a ausência de sobrecarga de volume.
  - Para pacientes em HD, pode-se incorporar a FRR no cálculo do Kt/V, atingindo um Kt/V semanal ≥ 2,1, embora essa abordagem seja pouco aplicada na prática clínica^2^. Além disso, dada a dificuldade de coleta de urina de 24 horas de forma confiável, principalmente em idosos, sugere-se, como alternativa, a dosagem de beta-2 microglobulina sérica para avaliação da FRR^14^.
- **Avaliação clínica**: presença de sintomas urêmicos, ganho de peso interdialítico (GPID), frequência de hospitalização, qualidade de vida e avaliação do estado nutricional.
- **Diurese residual**: o método mais simples para monitorar a diurese residual é solicitar ao paciente a mensuração da diurese de 24 horas uma vez por mês no próprio domicílio.

A diálise incremental tem potencial para retardar a perda da FRR, reduzindo a exposição à glicose na DP e as flutuações hemodinâmicas na HD. Entretanto, a FRR permanece vulnerável a múltiplos fatores agudos e crônicos, como uso de drogas nefrotóxicas, exposição a contraste, infecções e progressão da doença de base.

A prescrição da diálise incremental deve ser feita de maneira dinâmica e reavaliada periodicamente, preferencialmente em consultas mensais de rotina ou antes, quando houver necessidade. A monitorização da diálise incremental deve ser registrada em prontuário, incluindo justificativas explícitas para a manutenção do regime quando algum parâmetro estiver fora dos critérios de elegibilidade adotados, indicando possível ajuste e transição para a terapia convencional.

Medidas adjuvantes à diálise incremental podem incluir, quando pertinentes, a otimização do uso de diuréticos (preferencialmente de alça), bicarbonato de sódio por via oral e o uso de quelantes de fósforo e de potássio, além de dieta com restrição de potássio, fósforo e líquidos e adequada ingestão de proteínas^4^. O diurético de alça mais comumente utilizado, a furosemida, pode ser administrado em doses de até 240 mg ao dia. Embora doses superiores tenham sido relatadas, sua utilização deve ser criteriosa e acompanhada de monitorização adequada, devido ao risco aumentado de ototoxicidade^35,36,37,38^.

### Diferença Entre Diálise Incremental e Decremental

Enquanto a diálise incremental baseia-se em aumentar progressivamente a intensidade da diálise conforme a perda da FRR, a diálise decremental consiste na redução planejada da dose de diálise em situações de recuperação parcial da função renal após injúria renal aguda (IRA), melhora da função tardia do enxerto renal (DGF) ou no contexto de cuidados paliativos, quando o foco é alinhar o tratamento às metas de conforto e qualidade de vida^21^. Ambos os modelos — incremental e decremental — partem do mesmo princípio: personalizar a terapia e ajustar sua intensidade às necessidades dinâmicas do paciente. A análise comparativa das modalidades está descrita na Figura 1. Neste documento, abordaremos exclusivamente a diálise incremental.

**Figura 1:** Comparativo – diálise incremental e diálise decremental^8,9,21,31,39^.

Abreviaturas: FRR, Função Renal Residual; IRA, Injúria Renal Aguda; DGF, *Delayed Graft Function* (função tardia do enxerto).

### Critérios Para Transição Para a Modalidade de Diálise Convencional

A transição para a diálise convencional deve ocorrer quando não for possível manter os parâmetros clínicos e laboratoriais dentro das metas recomendadas, mesmo após ajustes progressivos da dose no regime incremental. As indicações estão resumidas no Quadro 2.

**Quadro 2:** Indicações de transição para diálise convencional

**Clínicos:**
Sinais/sintomas de hipervolemia e/ou ganho de peso entre as sessões > 5% do peso seco (no caso de hemodiálise).
Sintomas atribuídos à uremia.
Piora do estado nutricional.
Anúria.
Hospitalização frequente ou relacionada à hipervolemia ou uremia.
**Alterações laboratoriais persistentes por mais de três meses:**
Anemia refratária ao uso de eritropoetina e/ou à suplementação de ferro e atribuída à uremia.
Hiperfosfatemia e/ou hiperpotassemia refratárias ao manejo dietético e medicamentoso.
Acidose metabólica refratária.
Beta-2 microglobulina sérica (se disponível) persistente > 30 mg/L na ausência de infecção/inflamação.

Com o declínio da FRR, é necessário aumentar gradualmente a intensidade da terapia até atingir um esquema de dose plena:

- DP: Diálise Peritoneal Ambulatorial Contínua (DPAC), com quatro trocas, totalizando 8 L/dia, ou Diálise Peritoneal Contínua por Cicladora (DPCC) diariamente (ou seja, Diálise Peritoneal Automatizada – DPA no período noturno, somada à realização de troca manual ao longo do período diurno, totalizando 24 horas de diálise, todos os dias da semana);
- HD: três sessões semanais com duração de quatro horas cada.

Durante a transição da diálise incremental para a convencional, recomenda-se a adaptação progressiva da duração e da frequência das sessões, de modo a minimizar sintomas e desconfortos. Sessões de HD mais curtas (duas a três horas) podem ser adotadas temporariamente à medida que se aumenta a frequência, com ajustes adicionais conforme a resposta clínica.

Na Figura 2, apresenta-se um modelo esquemático da trajetória do paciente em DP incremental para o regime convencional. A Figura 3 ilustra a perspectiva da frequência do tratamento de HD em relação à taxa de filtração glomerular (TFG) e à FRR ao longo do tempo.

**Figura 2:** Modelo esquemático da trajetória do paciente em diálise peritoneal incremental.

Abreviaturas: FRR, Função Renal Residual; DPAC, Diálise Peritoneal Ambulatorial Contínua; DPA, Diálise Peritoneal Automatizada.

**Figura 3:** Perspectiva da necessidade de sessões semanais de HD de acordo com a função renal residual ao longo do tempo.

Abreviaturas: TFG, Taxa de Filtração Glomerular; HD, Hemodiálise.

Fonte: Adaptado de Murea e Kalantar-Zadeh^40^.

### Variações e Exemplos de Esquemas Mais Comuns de Diálise Incremental

Nesta seção, são apresentadas as principais variações e exemplos de esquemas de diálise incremental utilizados na prática clínica. Embora diferentes estratégias tenham sido propostas, ainda não existe consenso sobre o modelo ideal de diálise nesse contexto.

#### Cálculo da Dose de Diálise, Função Renal Residual

Desde 1998, modelos matemáticos vêm sendo desenvolvidos para mensurar a depuração renal e dialítica^41^. O cálculo baseia-se em modelos teóricos e exige a compreensão das diferentes taxas de depuração da ureia pela diálise e pelos rins^42^. Atualmente, quatro métodos principais são descritos para integrar a FRR ao cálculo da dose de diálise. Embora pouco utilizados na prática clínica, devido à sua complexidade, é importante conhecer seus fundamentos; a comparação entre eles encontra-se no Quadro 3. Dá-se mais ênfase aos métodos 1 e 2, que são métodos complementares mais utilizados na prática clínica. Além desses, há ainda um método de estimativa da FRR baseado exclusivamente em exames laboratoriais (beta-2 microglobulina, creatinina e ureia)^4^.

**Quadro 3:** Comparação dos métodos para cálculo e prescrição da diálise incremental

Aspecto	Método 1Diretriz KDOQI	Método 2Soma de Kt/V residual e dialítico	Método 3Modelagem UKM avançada (inclui UF)	Método 4SPEEDY
Como calcular	Se Kru ≥ 2 mL/min/1,73 m^2^ e estabilidade clínica/volêmica: pode-se considerar 2x/sem; intensificar conforme queda da FRR.	Depuração: depuração (mL/min) = [[ureia urinária × volume urinário] ÷ 1440] ÷ ureia plasmática média; converter para L/sem: mL/min × 10,08; estimar Krt/V e somar ao Kt/V dialítico.	Usar software de UKM para simular dose/tempo por sessão que atinja o alvo (stdKt/V ou EKRUN), incorporando UF e FRR contínua.	Usar planilha/app (SPEEDY) que, a partir de KRUn e frequência, calcula o Kt/V por sessão necessário para atingir o alvo semanal.
Vantagens	Simples, conservador, fácil adoção.	Flexível, personalizável, utiliza a FRR; metodologia familiar à DP/HD.	Teoricamente mais preciso; inclui UF e variáveis técnicas.	Prático e orientado à clínica; facilita personalização; integração digital (SPEEDY).
Limitações	Restritivo; não quantifica finamente a contribuição renal. Não se aplica à DP.	Requer coleta urinária acurada; depuração de ureia subestima TFG (margem de segurança); não inclui UF explicitamente.	Complexidade, necessidade de *software*, treinamento e dados detalhados. Não se aplica à DP.	Requer normatizações; manter monitorização clínica estreita. Não se aplica à DP.
Ferramentas	Diretrizes KDOQI; planilhas simples.	Coleta 24h; fórmula de *Watson* para V; calculadora *TouchCalc* Kt/V DP.	*Solute-Solver* e calculadoras de stdKt/V.	Planilha SPEEDY *(Spreadsheet for the Prescription of incrEmental haEmoDialYsis).*

Abreviaturas: FRR, função renal residual; Kru, depuração renal de ureia; KRUn, Kru normalizado; UF, ultrafiltração; stdKt/V, Kt/V padronizado semanal; Krt/V, componente residual do Kt/V; DP, diálise peritoneal; HD, hemodiálise.

##### • Método 1 (KDOQI)

Sugere o uso do Kru como ferramenta de monitoramento. O Kru reflete a capacidade de depuração renal remanescente, sendo uma das ferramentas recomendadas para o ajuste da dose de diálise incremental. Segundo as diretrizes do KDOQI, pacientes com Kru de 3 mL/min equivalem a um Kt/V semanal de aproximadamente 1,0. Portanto, pacientes com Kru ≥ 2 mL/min/1,73 m^2^ podem ser elegíveis para um esquema de HD com frequência inferior a três vezes por semana, visando a um stdKt/V semanal ≥ 2,1. Já aqueles em regime convencional de três sessões semanais devem alcançar spKt/V ≥ 1,2 por sessão^5^. Para o cálculo do Kru, pode-se utilizar a calculadora do *UpToDate* (https://www.uptodate.com/contents/calculator-residual-kidney-urea-clearance-kru-in-hemodialysis-patients) devido à sua praticidade, confiabilidade e disponibilidade, bem como à sua capacidade de incorporar variações do período de coleta de urina. O Kru depende do volume urinário, do tempo do período interdialítico e da concentração média de ureia (calculada pela soma das dosagens de ureia após a primeira e a segunda diálise da semana, dividida por dois). Como a fórmula da calculadora utiliza BUN (blood urea nitrogen), é necessária a conversão prévia dos valores de ureia para BUN.

##### • Método 2 (Kt/V)

Neste método, calcula-se o Kt/V semanal proveniente da FRR, o qual é então somado ao Kt/V semanal das sessões de diálise. A FRR é quantificada com base na depuração de ureia, prática utilizada tanto na DP quanto na HD. Embora a depuração de ureia subestime a verdadeira TFG, ela fornece uma margem de segurança adequada para decisões clínicas.

Uma das formas de estimar a depuração de ureia inclui^43^:

- A coleta da ureia plasmática antes e após duas sessões consecutivas de HD e o cálculo da média;
- A medição da diurese em 24 horas no intervalo interdialítico;
- A dosagem da concentração de ureia na urina de 24 horas.

A fórmula para o cálculo da depuração urinária de ureia é:

Depuração (mL/min) = [[ureia urinária (mg/dL) × volume urinário (mL/24h)] × 24 ÷ 1440] ÷ ureia plasmática média (mg/dL)

Uma vez calculada a depuração de ureia (Kru), pode-se estimar o Kt/V residual semanal (Krt/V), sendo Kr (L/semana) igual à depuração de ureia convertida de mL/min para L/semana. Como a depuração de ureia é obtida em mL/min, deve-se multiplicar por 10,08, a fim de converter essa unidade de medida para L/semana. O fator tempo (t) corresponde a 1 (semana), a contribuição residual pode ser expressa como Kru × 10.080 / V, e o V pode ser estimado utilizando a fórmula de *Watson*. O Kr mencionado corresponde ao Kru, diferindo apenas na unidade (L/semana vs. mL/min).

Outra possibilidade é obter o Krt/V por meio de uma calculadora online (https://www.zkidney.com/pd-formulas). Esta calculadora foi criada para uso na DP; portanto, nos casos de pacientes em HD incremental, o item “clearance do dialisato (dialysate clearance)” deve ser considerado zero^30,44,45,46^. O Kt/V standard representa a dose semanal de diálise e deve ser somado ao Kt/V proveniente da FRR semanal para obtenção do Kt/V total semanal. No site da SBN, uma calculadora para o cálculo de Kt/V *standard* encontra-se disponível (https://sbn.org.br/medicos/utilidades/calculadoras-nefrologicas/kt-v/).

Segue exemplo prático:

Paciente feminina, 40 anos, 74 kg, altura 165 cm.

• Ureia após a primeira diálise da semana: 38 mg/dL (≈ BUN 17,7 mg/dL)

• Ureia antes da segunda diálise da semana: 94 mg/dL (≈ BUN 43,9 mg/dL)

• Volume urinário interdialítico (44 horas/2640 min): 3550 mL

• Ureia urinária interdialítica: 191 mg/dL

Cálculo do KRU (via calculadora):

• Volume urinário: 3550 mL

• Concentração de ureia urinária: 191 mg/dL

• Período interdialítico: 2640 min

• BUN inicial: 17,7 mg/dL

• BUN final: 43,9 mg/dL

→ KRU = 7,87 mL/min

Cálculo do Kt/V renal semanal:

• K = 7,87 mL/min

• t = 10.080 min (1 semana)

• V (Watson) = 33,8 L = 33.800 mL

→ Kt/V renal = (7,87 × 10.080) / 33.800 = 2,34

Cálculo do Kt/V da diálise (semanal), que pode ser acessado pela calculadora da SBN:

• Ureia pré-diálise: 94 mg/dL

• Ureia pós-diálise: 27 mg/dL

→ Kt/Vsp = 1,37

→ Kt/Vstd = 1,42

Kt/V semanal total:

→ Kt/V total = 2,34 + 1,42 = 3,76

##### • Método 3 (*Urea Kinetic Model* – UKM)

Este é considerado o modelo mais teoricamente preciso, pois incorpora não apenas a depuração contínua pela FRR, mas também o impacto da ultrafiltração^42^. Baseia-se em equações matemáticas complexas que integram as diferentes formas de remoção de solutos (contínua pelos rins e intermitente pela máquina), além da eliminação de líquidos.

##### • Método 4 (*Spreadsheet for the Prescription of incrEmental haEmoDialYsis* – SPEEDY)

Mais recente, esse método parte do princípio de que a dose de diálise deve ser proporcional à FRR^47^. Nesse modelo, a FRR é ajustada ao número de sessões semanais, permitindo que pacientes com função renal mais preservada possam iniciar ou manter regimes com menor frequência dialítica. Desde que o somatório da depuração renal e dialítica atinja o Kt/V alvo, o tratamento é considerado adequado. Para isso, uma série de equações é utilizada, na maioria dos casos, idênticas às usadas pelo *Solute-Solver* que está disponível no site www.ureakinetics.org. O passo a passo da planilha encontra-se descrito em Casino e Basile^47^.

#### Prescrição de Diálise Incremental

Os modelos práticos de prescrição de HD e DP incrementais estão apresentados nos Quadros 4 e 5.

**Quadro 4:** Opções práticas para a hemodiálise incremental^4,8,9,21,31,39^

Estratégia	Como aplicar	Vantagens	Riscos/atenções
Baseada no Kru	Kru ≥ 2 mL/min e/ou diurese ≥ 500 mL/24h: **realizar HD 2x/semana** Kru ≥ 3,5 mL/min, paciente aderente e centro experiente: **realizar HD 1x/semana em casos selecionados**	Preservar FRR.	Subdiálise se FRR superestimada, hipervolemia, alta taxa de UF.
Redução do número de sessões	Iniciar 1 a 2x/semana quando há FRR substancial, estabilidade volêmica e adesão.	Preserva FRR; menor carga de tratamento.	Subdiálise se FRR superestimada; alto ganho interdialítico.
Redução do tempo de sessão	2 a 3h por sessão, mantendo 2 a 3x/semana.	Menor fadiga; logística facilitada.	Menor depuração por sessão; maior taxa de UF.
Redução de fluxos de sangue (FS) e dialisato (FD)*	FS 200 a 250 mL/min; FD 300 a 400 mL/min.	Menor eficiência e menos sintomas de desequilíbrio.	Redução do Kt/V por sessão; atenção à recirculação.
Redução da área de superfície do filtro de HD (dialisador)*	Dialisador de HD com menor área de superfície.	Redução da dose de diálise.	Subdiálise.
Combinações	Combinar 2 a 3 medidas acima, com metas explícitas.	Personalização fina.	Maior complexidade de monitorização.

Abreviaturas: FRR, função renal residual; UF, ultrafiltração; FS, fluxo de sangue; FD, fluxo de dialisato; HD, hemodiálise.

Nota: * A redução do FD, do FS ou da área de superfície do dialisador é uma medida complementar e deve ser empregada com extrema cautela, jamais como substituto da dose dialítica mínima ou como prática de racionalização de custos (no caso do filtro de HD e do FD). Essas estratégias só são apropriadas quando acompanhadas de FRR mensurada, com critérios de elegibilidade atendidos e com monitoramento seriado da adequação. Seu uso indevido descaracteriza a abordagem incremental e contraria este posicionamento.

**Quadro 5:** Prescrições incrementais em diálise peritoneal^34,49,90,91,92^

Modalidade	Exemplos de prescrição inicial	Situações clínicas e justificativas	Sinais de perda de FRR ou inadequação	Ajustes progressivos recomendados
DPAC (Diálise Peritoneal Ambulatorial Contínua)	– 2–3 trocas/dia de 2 L; – 1 troca/dia de icodextrina (8–16h); – 3 trocas de 1,5–2 L por 4–6 dias/semana.	– Pacientes com diurese >100 mL/dia; – Estilo de vida ativo; – Menor exposição à glicose e à sobrecarga mecânica.	– Aumento de ureia, creatinina e fósforo; – Hipervolemia (edema, ganho de peso); – Redução da diurese; – Sintomas urêmicos.	– Aumentar para 3–4 trocas/dia; – Usar volumes de 2–2,5 L, conforme tolerância; – Adicionar icodextrina noturna; – Passar para 7 dias/semana.
DPA (Diálise Peritoneal Automatizada)	– Dias sem diálise (3–5 noites/semana); – Menor volume de infusão (4–5 ciclos noturnos com 1,2–1,5 L); – Sem diálise durante o dia (DP noturna intermitente; dia seco); – Menos tempo conectado à cicladora (sessões de 6–7h, 8–10 L totais).	– Maior liberdade para trabalho, estudo e atividades sociais; – Boa FRR para compensar ausência de permanência diurna; – Menos tempo conectado à cicladora.	– Redução da remoção de solutos; – Hipervolemia; – Queda acentuada da diurese.	– Aumentar volume por ciclo (até 2–2,5 L); – Aumentar número de ciclos (até 5–6); – Alterar para CCPD; – Expandir para 7 noites/semana.
Icodextrina (isolada ou associada)	– 1 troca longa/dia (8–12h).	– Pacientes com FRR preservada; – Indicado para controle de volume sem glicose.	– Redução da diurese; – Hipervolemia ou sintomas urêmicos.	– Utilizar em troca longa diurna ou noturna, conforme modalidade, combinada com trocas adicionais de soluções à base de glicose.

Abreviaturas: FRR, função renal residual; DP diálise peritoneal; CCPD, diálise peritoneal cíclica contínua (*Continuous Cycling Peritoneal Dialysis*).

### Vantagens e Desfechos da Diálise Incremental em Comparação à Convencional

#### Hemodiálise

No contexto do cuidado de pacientes submetidos à diálise, a FRR não é apenas uma variável utilizada para a indicação e o monitoramento do tratamento, mas também uma métrica de desfecho a ser mantida. O crescimento da evidência quanto à manutenção de um *clearance* renal e seu impacto na sobrevida de pacientes com DRC-5D é robusto, com a publicação de dados do NECOSAD, em 2004, inicialmente mostrando esse benefício^25^, uma associação que tem sido corroborada ao longo dos anos^18,26,48^.

Ao analisar o impacto da HD incremental sobre a preservação da FRR, alguns pontos podem ser destacados como mecanismos simbióticos para sua ocorrência, tais como: (a) maior excreção de sódio e água, levando a (b) melhor controle volêmico, (c) menor risco de hipotensão intradialítica e (d) menor ocorrência de eventos isquêmicos renais^49^. Essa evidência de manutenção de *clearance* renal é observada em coortes de estudos observacionais, com preservação do débito urinário, bem como do *clearance* de ureia e de creatinina nos grupos que realizaram HD com menor frequência (uma ou duas vezes na semana)^18,50,51,52^.

O uso de esquemas de HD incremental deve ser ponderado na adaptação do paciente e de seus cuidadores durante a transição do cuidado ao iniciar a HD. A carga de tempo, sintomas e o contexto de estresse psíquico dessa mudança não devem ser minimizados, pois trazem grande impacto na vida desses indivíduos. Nesse cenário, o ensaio piloto TWOPLUS, conduzido em pacientes incidentes com FRR (diurese ≥ 500 mL/dia e TFG ≥ 5 mL/min/1,73 m^2^), comparou o início incremental *versus* HD em esquema convencional. Os pacientes com início incremental apresentaram menor carga de sintomas psicológicos, menor fadiga e maior sensação de autonomia^53^.

Além dos benefícios clínicos, a HD incremental tem potencial de reduzir a carga ambiental ao diminuir a frequência semanal de sessões. Cada sessão de HD em centro é ambientalmente ofensora, com uma média de 58,9 kg CO_2_-eq por tratamento, além do consumo de água, com estimativas que indicam o uso de 265 milhões de m^3^/ano. Dessa forma, a menor frequência de sessões também reduz a captação e o descarte hídrico semanais, aliviando pressões sobre recursos locais^54,55^. É importante reconhecer que faltam estudos que comparem diretamente a pegada de carbono de programas incrementais *versus* convencionais^56^. Esse argumento alinha-se com a chamada do KDIGO por um tratamento renal sustentável^54^ e com as preocupações da ANVISA sobre o uso sustentável de recursos em serviços de saúde renal.

Com relação aos desfechos clínicos, em especial ao evento de hospitalização em pacientes em HD, há evidência de uma metanálise com 36 estudos envolvendo 138.939 pacientes. Essa análise comparou esquemas de HD realizados uma a duas vezes por semana com HD três vezes por semana, demonstrando menor ocorrência do desfecho de hospitalizações no grupo incremental. Na avaliação de potenciais complicações decorrentes da “menor oferta” de diálise, não foram encontradas diferenças estatisticamente significativas entre os grupos quanto aos eventos de hiperpotassemia e disfunção de acesso vascular^24^. Outros estudos observacionais sugerem redução das hospitalizações^48,57^.

No seguimento de pacientes em HD incremental, é importante considerar o potencial impacto dessa estratégia na sobrevida, em comparação à HD convencional. Metanálises demonstram que a HD incremental não se associa ao aumento significativo da mortalidade, em comparação à HD convencional, quando aplicada em pacientes adequadamente selecionados^15,24^. De forma semelhante, em um ensaio clínico não randomizado, multicêntrico, envolvendo 132 pacientes em HD três vezes por semana e 71 pacientes em HD duas vezes por semana, não houve diferença na sobrevida entre os grupos após 24 meses de acompanhamento^58^. Em uma análise secundária do BISTRO *trial*, um ensaio clínico randomizado, pacientes com maior TFG ao iniciar a terapia dialítica seguiram com maior sobrevida no longo prazo^59^.

#### Hemodiafiltração (HDF)

A evidência quanto ao uso de HDF em uma modalidade incremental apresenta plausibilidade mecanista, devido à maior remoção de moléculas médias quando a dose convectiva adequada é atingida durante a sessão^60^. Uma série de casos, incluindo 11 pacientes incidentes em diálise, avaliou o uso de HDF uma vez por semana no seguimento, com um escalonamento da terapia para duas a três vezes por semana em uma mediana de tempo de 7 meses (IIQ 3–24 meses)^61^. Entretanto, até o momento da publicação deste posicionamento, ainda carecemos de evidências comparando seu uso e seus benefícios em relação à prescrição convencional de HDF ou mesmo de HD como controles.

#### Diálise Peritoneal

Os relatos iniciais sobre DP incremental concentraram-se em demonstrar a viabilidade da técnica e sua segurança clínica, utilizando metas baseadas em Kt/V. Burkart e Satko, em 2000, descreveram um pequeno grupo de 13 pacientes em DPAC acompanhados por dois anos, confirmando a possibilidade de manter o tratamento incremental com parâmetros adequados de depuração^62^. No mesmo ano, De Vecchi et al.^63^ seguiram 25 pacientes tratados inicialmente com uma a duas trocas diárias, observando baixas taxas de peritonite e maior aceitação do regime reduzido de trocas quando comparado aos esquemas tradicionais de três a quatro trocas.

Embora concebida inicialmente como dependente da FRR para viabilizar a prescrição, evidências posteriores sugerem que a abordagem incremental não apenas se beneficia da FRR, mas pode contribuir para sua preservação, incluindo manutenção de diurese, depuração e menor declínio ao longo do tempo^64,65,66,67,68,69,70,71^.

A sobrevida da técnica surge como um parâmetro favoravelmente associado à DP incremental. Fernandes et al.^70^ demonstraram maior sobrevida técnica em pacientes tratados de forma incremental. Resultados convergentes foram identificados por Sakurada et al.^72^, em análise de registro de pacientes adultos incidentes em DP entre 2007 e 2017, revelando menor necessidade de transferência precoce para HD no primeiro mês de terapia. Um potencial mecanismo explicativo reside na menor exposição a soluções peritoneais bioincompatíveis, em razão do menor número de trocas e da menor carga cumulativa de glicose, osmolaridade e acidez.

A melhora da qualidade de vida é um dos objetivos centrais da DP incremental, em virtude de um menor número de procedimentos e de menor sobrecarga terapêutica. Naljayan et al., em um estudo retrospectivo norte-americano com pacientes em DPAC ^73^, identificaram melhores escores no questionário *Kidney Disease Quality of Life* (KDQOL), particularmente nos domínios de componente físico, carga da doença renal e efeitos da doença renal. Em relação à carga procedimental, o menor número de conexões e desconexões sugere benefício operacional.

Do ponto de vista de segurança infecciosa, quatro estudos relataram menor risco de peritonite associado à DP incremental^64,66,72,74^. Contudo, achados mais recentes, oriundos de uma grande coorte multicêntrica do PDOPPS, acompanhando pacientes incidentes entre 2014 e 2017 em modalidade incremental ou convencional, não demonstraram diferenças significativas nesse desfecho^75^.

Os estudos que avaliaram hospitalização^66,70,74,76^ demonstraram associação entre a DP incremental e menores taxas de internação, inclusive entre pacientes com *diabetes mellitus* (DM)^74^. Esses achados reforçam o potencial da abordagem incremental em reduzir complicações e internações hospitalares, possivelmente relacionadas à manutenção da FRR, à menor exposição a soluções bioincompatíveis e à melhor experiência terapêutica.

Os resultados quanto à sobrevida do paciente são encorajadores. Lee^28^, comparando 232 pacientes em DP convencional e 71 em incremental, observou sobrevida semelhante entre os grupos, com vantagem significativa entre os pacientes com DM tratados de forma incremental. Liu et al.^69^, avaliando 285 pacientes incidentes em DPAC incremental *versus* 502 em DPAC plena, demonstraram redução de 39% no risco de óbito por todas as causas e de 41% de redução no risco de mortalidade nos primeiros seis anos. Yan et al.^74^, em coorte retrospectiva canadense com 175 pacientes, relataram baixa taxa de peritonite e de hospitalização, ainda que um maior volume inicial de infusão tenha se associado à maior mortalidade. Fernandes et al.^70^, em coorte portuguesa, também reportaram maior sobrevida associada à técnica incremental. Finalmente, resultados de centros do PDOPPS não demonstraram diferença na mortalidade dos pacientes^75^.

Apesar do crescente corpo de evidências observacionais, apenas um ensaio clínico randomizado controlado foi identificado. Yan et al.^77^ compararam 70 pacientes em DPAC com três trocas diárias *versus* 69 pacientes com quatro trocas, acompanhados por 24 meses, sem diferenças entre os grupos na sobrevida do paciente ou da técnica^28^. No campo das revisões sistemáticas, Garofalo et al.^15^ analisaram 22 estudos de DP e HD incremental, sendo sete especificamente de DP incremental, não encontrando diferenças significativas em FRR, sobrevida do paciente, sobrevida técnica ou tempo até escalonamento para dose completa após 24 meses. Xu et al.^28^, avaliando dez publicações, confirmaram desfechos comparáveis entre DP incremental e DP convencional em pacientes incidentes, sem diferenças em mortalidade, peritonite ou sobrevida técnica.

Em síntese, o corpo de literatura disponível, ainda que majoritariamente composto por estudos observacionais, aponta para a viabilidade e a potencial eficácia da DP incremental, sugerindo benefícios relacionados à manutenção da FRR, a menores taxas de hospitalização e à possível melhora da qualidade de vida, sem evidências consistentes de aumento de risco quando comparada à DP convencional.

### Desafios, Organização e Aspectos Legais

Embora conceitualmente simples, a implementação da diálise incremental envolve uma série de desafios organizacionais, logísticos e regulatórios. Por se tratar de um modelo de prescrição individualizada e dinâmica, que depende do acompanhamento sistemático da FRR e da adaptação progressiva da dose dialítica, sua aplicação requer a integração entre equipe multiprofissional, protocolos bem estruturados e suporte institucional. Além disso, aspectos legais e administrativos tornam-se relevantes, uma vez que a diálise incremental ainda não está amplamente reconhecida em alguns sistemas de saúde como modalidade específica de terapia renal substitutiva, o que pode gerar impasses relacionados à autorização, ao reembolso e ao monitoramento. Discutir esses elementos é essencial para viabilizar a expansão segura e sustentável da diálise incremental e para garantir equidade de acesso a essa estratégia promissora de início de diálise.

#### Riscos da Subdiálise e Resistência à Transição

Os riscos clínicos da subdiálise incluem sobrecarga volêmica, hiperfosfatemia, hiperpotassemia e acidose metabólica. A mitigação desses riscos depende de monitorização rigorosa por meio de exames laboratoriais seriados e avaliação clínica sistemática, com acompanhamento do volume urinário. Além disso, taxas de ultrafiltração elevadas em sessões espaçadas representam uma preocupação adicional, especialmente em esquema de uma sessão semanal^78^.

A literatura sobre resistência do paciente à transição da diálise incremental para o regime convencional ainda é escassa. Protocolos de implementação recentes incorporam medidas de aceitabilidade e desfechos relatados pelo paciente^79^, reconhecendo que a percepção subjetiva de bem-estar pode interferir na adesão ao escalonamento da dose dialítica. Para lidar com essa resistência, recomenda-se definir previamente critérios objetivos para intensificação da terapia, conforme exemplificado no Quadro 2. A revisão periódica do plano terapêutico, aliada à integração de estratégias de suporte – incluindo orientação nutricional, controle de volume e manejo de potássio e fósforo – favorece uma transição mais segura e menos traumática para o paciente^78,79^.

#### Organização do Centro de Diálise e Papel da Equipe Multidisciplinar

A implementação da diálise incremental requer uma estrutura organizacional adaptada para garantir segurança, efetividade e otimização de vagas de diálise. A experiência de centros que adotaram esse modelo evidencia que o planejamento estratégico e a padronização de protocolos são fundamentais para a sustentabilidade da prática. Um exemplo prático de como os centros de HD podem se organizar para otimização de vagas e recursos está apresentado no Quadro 6.

**Quadro 6:** Sugestão de organização de centros de hemodiálise para otimização de vagas em diálise incremental (1–2 sessões/semana)

Segunda-feira	Terça-feira	Quarta-feira	Quinta-feira	Sexta-feira	Sábado
Paciente 1 2x/semana	Paciente 2 2x/semana	Paciente 3 2x/semana	Paciente 1 2x/semana	Paciente 2 2x/semana	Paciente 3 2x/semana
Paciente 4 2x/semana	Paciente 5 2x/semana	Paciente 6 1x/semana	Paciente 7 1x/semana	Paciente 4 2x/semana	Paciente 5 2x/semana

Desde a admissão, é fundamental que o centro disponha de um plano de escalonamento previamente definido, com critérios objetivos para progressão da terapia (Quadro 2). A agenda assistencial deve ser planejada de forma flexível, prevendo sessões em frequência reduzida (uma a duas vezes por semana) e possibilitando rápida transição para três vezes por semana, conforme necessário^40,80,81^.

A rotina assistencial deve contemplar monitorização contínua e sistematizada, incluindo registro da diurese, dosagem seriada de exames laboratoriais em intervalos predefinidos, avaliação periódica da FRR e auditoria de eventos adversos, como hospitalizações^40,82^, de acordo com o Quadro 1.

Recomenda-se a adoção de painéis de indicadores de desempenho clínico que incluam não apenas parâmetros objetivos — como declínio da FRR, exames laboratoriais e sobrecarga volêmica —, mas também desfechos centrados no paciente, como fadiga, prurido e preferências individuais, para avaliar a necessidade de escalonamento não programado^79,81,83^.

A efetividade da estratégia depende de uma equipe multiprofissional integrada, com atribuições bem definidas entre o nefrologista^80^, a equipe de enfermagem^82^, a nutrição^84^, a farmácia clínica^79^ e as equipes do serviço social e de psicologia^85,86^, em relação a: (a) educação do paciente e da família; (b) acompanhamento nutricional e dietético; (c) monitoramento da diurese e dos parâmetros clínicos e laboratoriais; e (d) padronização e atualização de protocolos institucionais. Vale ressaltar que programas estruturados de educação pré-diálise, com participação ativa da equipe multiprofissional, podem melhorar a transição do cuidado e favorecer escolhas informadas mais assertivas^84^.

#### Aspectos Legais, Éticos e de Financiamento

Embora as principais diretrizes reconheçam a diálise incremental como uma estratégia válida e importante de início de diálise, as orientações detalhadas sobre sua aplicação prática ainda são limitadas, contribuindo para a heterogeneidade entre países e centros de diálise^4^. No Brasil, a diálise é predominantemente financiada pelo Sistema Único de Saúde (SUS), e modalidades além da convencional são geralmente restritas a pacientes com planos privados. A personalização por meio da diálise incremental pode reduzir custos ao diminuir o consumo de água, de insumos e de recursos de equipe assistencial^4^. Estimativas sugerem que, se implementada amplamente nos Estados Unidos, poderiam ser economizados de 250 a 500 milhões de dólares anuais pelo *Medicare*. Atualmente, no Brasil, não há remuneração específica para a diálise incremental, nem incentivos para modalidades de diálise mais sustentáveis e/ou centradas no paciente^87^.

Do ponto de vista ético, estudos reportaram segurança e aceitabilidade da abordagem incremental^12^. A base dessa estratégia é a decisão compartilhada, envolvendo médicos, equipe multidisciplinar, pacientes e familiares. Essa abordagem pressupõe corresponsabilidade no monitoramento de sintomas, na adesão ao tratamento e na revisão contínua das metas terapêuticas. Os benefícios e os riscos da diálise incremental devem ser avaliados de forma individualizada, garantindo que a prescrição dialítica respeite não apenas os critérios clínicos, mas também os valores, preferências e a qualidade de vida de cada paciente^88^.

A diálise incremental é uma alternativa viável, segura e personalizada para pacientes com FRR preservada. Sua adoção, entretanto, ainda enfrenta barreiras regulatórias, legais e de financiamento. A consolidação dessa prática requer atualização das diretrizes nacionais, revisão dos modelos de custeio, fortalecimento do papel das equipes multiprofissionais e participação ativa dos pacientes nas decisões terapêuticas.

### Perspectivas do Paciente

A doença renal crônica em estágio final (DRC-5D) impõe desafios consideráveis à vida dos pacientes, os quais frequentemente se estendem muito além das limitações clínicas. O tratamento dialítico convencional, com sua rotina rígida e intensiva, pode representar um novo peso sobre indivíduos que já vinham convivendo com restrições severas durante a fase conservadora da doença. Nesse contexto, a experiência pré-dialítica costuma ser marcada por restrições alimentares e hídricas rigorosas, medo de descompensações e sensação de perda de autonomia. Muitos pacientes descrevem esse período como uma existência controlada, permeada pela ansiedade de “errar” e sobrecarregar os rins. Paradoxalmente, o início da diálise passa a ser visto não apenas como necessidade médica, mas como uma possível libertação das restrições anteriores. É justamente nesse ponto que a diálise incremental surge como uma alternativa transformadora. Diferente do modelo rígido de início imediato em dose plena, a diálise incremental propõe uma transição mais humana, que respeita a FRR do paciente e ajusta o tratamento de forma gradual^14,17,34,70,89^. Essa abordagem promove uma adaptação menos traumática, reduz a sobrecarga física e emocional e devolve ao paciente parte da liberdade e da qualidade de vida perdida.

Tradicionalmente, ao chegar à diálise, o caminho do paciente costuma ser o início direto em regime completo da terapia – três sessões semanais de HD ou o equivalente em DP. Porém, muitos ainda possuem FRR, capaz de contribuir para o equilíbrio metabólico. A diálise incremental valoriza esse potencial, complementando de forma suave aquilo que o organismo ainda consegue realizar e aumentando gradualmente a dose conforme a FRR diminui^17,34^.

Do ponto de vista do paciente, os principais benefícios são sentidos em diferentes dimensões:

- Menos restrições, mais qualidade de vida: ao preservar a FRR, a diálise incremental permite maior flexibilidade nutricional e hídrica. Isso significa comer com menos medo, beber com menos culpa e viver com mais autonomia. Para o paciente que passou meses ou anos sob um regime conservador restritivo, esse é um ganho imensurável.
- Adaptação gradual à diálise: o início da diálise é um marco emocional duro. A possibilidade de começar com um regime mais leve faz com que a adaptação seja menos traumática, diminuindo a sensação de perda de controle e aumentando a resiliência psicológica^31^.
- Tempo livre e autonomia: em vez de estar inserido desde o início em um cronograma exaustivo de sessões, o paciente mantém mais espaço em sua rotina. Isso preserva não apenas a vida social e profissional, mas também a sensação de que a doença não tomou completamente seu cotidiano.
- Preparo para o futuro: nos casos em que a progressão da doença torna inevitável a necessidade de diálise plena, a diálise incremental já funciona como um período de treinamento. O paciente se adapta gradualmente à nova realidade, sem rupturas bruscas, o que melhora a adesão e os resultados clínicos.

A diálise incremental transcende o aspecto técnico da prescrição dialítica: ela representa uma mudança de paradigma ao reconhecer o paciente como protagonista do próprio tratamento. Ao integrar ciência e empatia, essa abordagem preserva a FRR, reduz a carga terapêutica, favorece o equilíbrio nutricional e metabólico, melhora o bem-estar psicológico e promove uma progressão gradual até a necessidade de diálise plena.

Mais do que prolongar a vida, ela oferece condições para que essa vida seja vivida com mais plenitude.

### Recomendações da SBN Sobre como Realizar a Diálise Incremental

Com base nas evidências científicas e na opinião de especialistas apresentadas ao longo deste posicionamento, a SBN recomenda a implementação de HD e DP incrementais, conforme resumido no Quadro 7. Para facilitar a aplicação prática das recomendações, incluímos um fluxograma decisório (Figura 4) que resume, de forma objetiva, as etapas para a implementação da diálise incremental.

**Quadro 7:** Recomendações da SBN e orientações para a realização da diálise incremental

Recomendações	Como fazer
Implementação	Realizar protocolo institucional de acordo com a realidade local.
Equipe multiprofissional capacitada	Capacitar médicos, equipe de enfermagem, nutricionistas, psicólogos e assistentes sociais sobre a diálise incremental, para auxiliar no sucesso da terapia.
Avaliação dos critérios de inclusão de paciente em diálise incremental	HD ≥ 500 mL de diurese; DP ≥ 100 mL de diurese; Kru ≥ 2 mL/min/1,73 m^2^; Ausência de hipervolemia e hipertensão arterial não controlada; Ausência de sintomas urêmicos; Controle adequado do metabolismo mineral e ósseo; Controle adequado do potássio ( ≤ 5,0 mEq/L); Controle adequado do estado volêmico (ganho de peso interdialítico < 5% do peso seco); Adesão do paciente ao tratamento e capacidade de monitoramento; Hospitalização infrequente e não relacionada à prescrição de diálise; Ausência de anemia persistente e de difícil controle; Bom estado nutricional, sem estado hipercatabólico acentuado.
Decisão compartilhada	Realizar decisão compartilhada com o paciente, seus familiares e a equipe médica e multidisciplinar.
Educação do paciente e da família sobre a terapia incremental	Orientar o paciente sobre a necessidade de monitorização urinária, coleta de exames, dieta, medicações e sobre a necessidade de evolução para terapia convencional quando indicado.
Prescrição de medicações que aumentam a FRR e auxiliam no controle da diálise incremental	Diuréticos, especialmente de alça; Bicarbonato de sódio via oral; Quelantes de fósforo; Quelantes de potássio.
Orientação da dieta por nutricionista	Dieta hipossódica; Restrição de potássio e fósforo; Adequada ingestão de proteínas.
Monitoramento do paciente em diálise incremental	Os parâmetros que precisam ser monitorados são: – Exames laboratoriais (hemograma, potássio, gasometria venosa, fósforo, paratormônio); – Avaliação clínica em todas as sessões e consultas (hipervolemia, uremia); – Diurese residual; Ver detalhes e periodicidade no Quadro 1.
Critérios para intensificação da diálise	**Clínicos:** sinais de hipervolemia, sintomas atribuídos à uremia, desnutrição, anúria, hospitalização por hipervolemia ou uremia. **Laboratoriais:** anemia refratária, distúrbios do metabolismo mineral e ósseo, hiperpotassemia ou acidose metabólica refratária. Ver detalhes no Quadro 2.
Registro e documentação sistemática	Documentar o início da terapia, os critérios, a evolução clínica e laboratorial e os ajustes, com justificativas, mensalmente.
Avaliação de desfecho clínico	Avaliar desfechos como hospitalização, complicações, diurese residual e transição para terapia convencional.

Abreviaturas: HD, hemodiálise; DP, diálise peritoneal. Nota: Apesar de existirem ensaios clínicos randomizados e metanálises sugerindo sobrevida semelhante entre a diálise incremental e a diálise convencional, as recomendações desta diretriz da SBN fundamentam-se nas evidências atualmente disponíveis — predominantemente observacionais — e no consenso de especialistas, com foco em padronização, segurança e aplicabilidade ao contexto brasileiro.

**Figura 4:** Fluxograma decisório para diálise incremental.

## Conclusão

A diálise incremental representa uma estratégia racional, segura e baseada em evidências para o início da terapia dialítica. Estudos demonstram que ela contribui para a preservação da FRR, reduz a exposição a riscos inerentes à diálise convencional e melhora a qualidade de vida. Quando aplicada a pacientes criteriosamente selecionados e acompanhada por monitoramento adequado, a abordagem incremental não se associa a maior mortalidade nem ao aumento de complicações.

Além dos benefícios clínicos, a estratégia colabora com a sustentabilidade do sistema de saúde, ao otimizar o uso de recursos sem comprometer a efetividade do tratamento. Trata-se de uma estratégia que favorece um modelo de cuidado individualizado e centrado no paciente – valores que se alinham aos princípios do cuidado contemporâneo.

Diante desse cenário, a SBN reafirma que a diálise incremental constitui uma alternativa válida, segura e sustentável, que deve ser incorporada de maneira estruturada aos centros de diálise, com critérios claros de elegibilidade, monitoramento e transição terapêutica.

## Data Availability

Não há dados adicionais para serem disponibilizados.
